# Lunar soil record of atmosphere loss over eons

**DOI:** 10.1126/sciadv.adm7074

**Published:** 2024-08-02

**Authors:** Nicole X. Nie, Nicolas Dauphas, Zhe J. Zhang, Timo Hopp, Menelaos Sarantos

**Affiliations:** ^1^ Origins Laboratory and Department of the Geophysical Sciences and Enrico Fermi Institute, The University of Chicago, Chicago, IL 60637, USA.; ^2^ Department of Earth, Atmospheric, and Planetary Sciences, Massachusetts Institute of Technology, Cambridge, MA 02139, USA.; ^3^ Max Planck Institute for Solar System Research, 37077 Göttingen, Germany.; ^4^ Heliophysics Science Division, NASA Goddard Space Flight Center, Greenbelt, MD 20771, USA.

## Abstract

The Moon has a tenuous atmosphere produced by space weathering. The short-lived nature of the atoms surrounding the Moon necessitates continuous replenishment from lunar regolith through mechanisms such as micrometeorite impacts, ion sputtering, and photon-stimulated desorption. Despite advances, previous remote sensing and space mission data have not conclusively disentangled the contributions of these processes. Using high-precision potassium (K) and rubidium (Rb) isotopic analyses of lunar soils from the Apollo missions, our study sheds light on the lunar surface-atmosphere evolution over billions of years. The observed correlation between K and Rb isotopic ratios (δ ^87^Rb = 0.17 δ ^41^K) indicates that, over long timescales, micrometeorite impact vaporization is the primary source of atoms in the lunar atmosphere.

## Teaser

Meteorite impacts supply atoms to the lunar atmosphere.

## INTRODUCTION

Space weathering plays a major role in shaping the surfaces of planetary bodies. One major consequence of space weathering is the production of tenuous, collisionless, gravitationally bound atmospheres (exospheres) surrounding planetary bodies such as the Moon and Mercury. Studying these atmospheres can provide valuable insights into how space weathering operates (*1*, *2*). For the Moon, considerable effort has been dedicated to mapping the spatial distribution and tracking the temporal evolution of alkali metal elements Na and K in the lunar atmosphere (*3*–*7*), including through the recent Lunar Atmosphere Dust and Environment Explorer (LADEE) space mission (*8*, *9*).

**Fig. 1.**
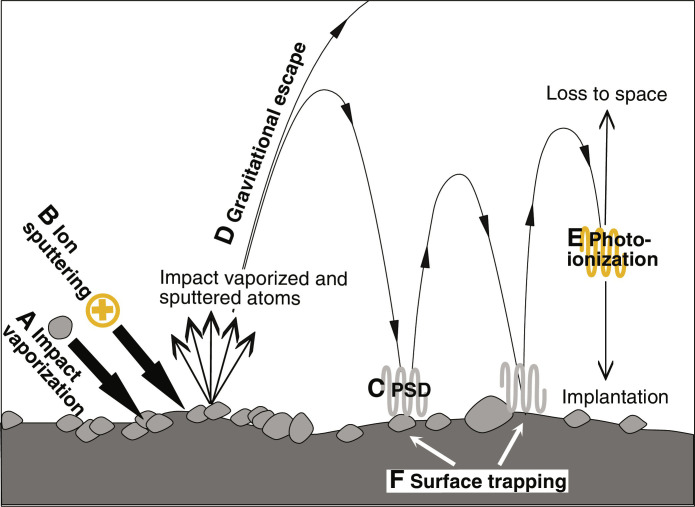
Possible lunar atmospheric sources and sinks. The sources include (**A**) meteoroid impact vaporization (IV), (**B**) solar wind ion sputtering (IS), and (**C**) photon-stimulated desorption (PSD). Impact vaporization and solar wind ion sputtering liberate atoms from rocks, while PSD only releases weakly bound adsorbed atoms. Once atoms are released by IS or IV, a fraction of them are lost to space through (**D**) gravitational escape. PSD does not cause any gravitational escape due to its low energy. The atoms that do not directly escape can hop multiple times on the lunar surface until they are eventually lost to space or reimplanted onto the lunar surface by (**E**) photoionization or are (**F**) permanently trapped on the lunar surface.

In the low-density lunar atmosphere, atoms hop on ballistic trajectories until they are either returned permanently to the surface, or lost to space (Fig. 1). Loss of atoms from the atmosphere can happen by photoionization, whereby solar photons ionize neutral atmospheric atoms that are either swept away by the solar wind or implanted on the lunar surface. Neutral atoms can also be trapped permanently in shadows on the lunar surface (surface trapping). In addition, some atoms can gain sufficient energy when they are released from the lunar surface to directly escape to space (gravitational escape). Photoionization, surface trapping, and gravitational escape represent the three major sinks of lunar atmosphere atoms, and they are balanced by three possible sources that comprise vaporization by micrometeoroid impacts (IV), ion sputtering (IS) of lunar regolith by solar wind charged particles, and photon-stimulated desorption (PSD) of surface atoms by solar ultraviolet photons (Fig. 1) (*6*). Associating observed alkali elements in the lunar atmosphere with their sources is challenging because the atmosphere is dominated by recycled atoms that bounce multiple times on lunar surface, and only a small fraction (<10%) are primary atoms freshly released from the lunar surface (*10*). Recent observations by the LADEE orbiter seem to indicate a substantial influence of meteorite impact vaporization and surface composition on the atmospheric composition, at least during meteor showers, but solar wind activity also seems to play an important role (*8*, *9*, *11*).

Isotopic analyses of alkali elements have the potential to help identify the origin of the lunar atmosphere because different sources can fractionate isotopes in different manners. Specifically, the isotopic fractionations of K and Rb are highly sensitive to the mechanisms that sustain the lunar atmosphere. This is because K and Rb are susceptible to vaporization during micrometeoroid impacts, ejection by ion sputtering, and desorption from lunar surface by solar photons. Lithium is less volatile (*12*), and Na and Cs have only one stable isotope each, so these alkali elements are less useful for our purpose. The lunar atmosphere is too tenuous for its isotopic composition to be directly analyzed, but lunar soils represent a reservoir that is shaped by interactions with the lunar atmosphere over billions of years (detailed modeling on the interactions between them can be found in the Supplementary Materials). We report here analyses of the isotopic compositions of K and Rb in 10 lunar samples (one lunar basalt and nine lunar soil samples; data S1A), returned by the Apollo missions from five distinct landing sites. The lunar basalt was analyzed to evaluate data accuracy, as previous studies showed that limited K and Rb isotopic fractionations were present in igneous rocks that experienced little space weathering (*13*–*17*). We selected soil samples that were previously identified to have isotopically fractionated K (*13*, *18*–*20*). For Rb isotopes, the only previous study on lunar soils had insufficient precision (~±1.7‰) to discern any isotopic variations (fig. S1) (*18*).

## RESULTS

We purified K and Rb from acid-digested aliquots of homogenized sample powders, using a method that allows to extract the two elements from a single sample simultaneously and quantitatively (*14*, *21*). We measured the K and Rb isotopic compositions using multicollector–inductively coupled plasma mass spectrometry (MC-ICPMS), with an overall precision of ~ ± 0.05‰ [95% confidence interval (CI)] for both elements (data S1A). The isotopic compositions are presented using the conventional delta (δ) notation, which represents the per mil (‰) difference in the measured isotopic ratio of a sample relative to a reference standard (SRM3141a for K and SRM984 for Rb)

δK 41 (‰)=[(K 41/K 39)sample/(K 41/K 39)SRM3141a−1]×1000

δRb  87(‰)=[(Rb 87/Rb 85)sample/(Rb 87/Rb 85)SRM984−1]×1000

**Fig. 2.**
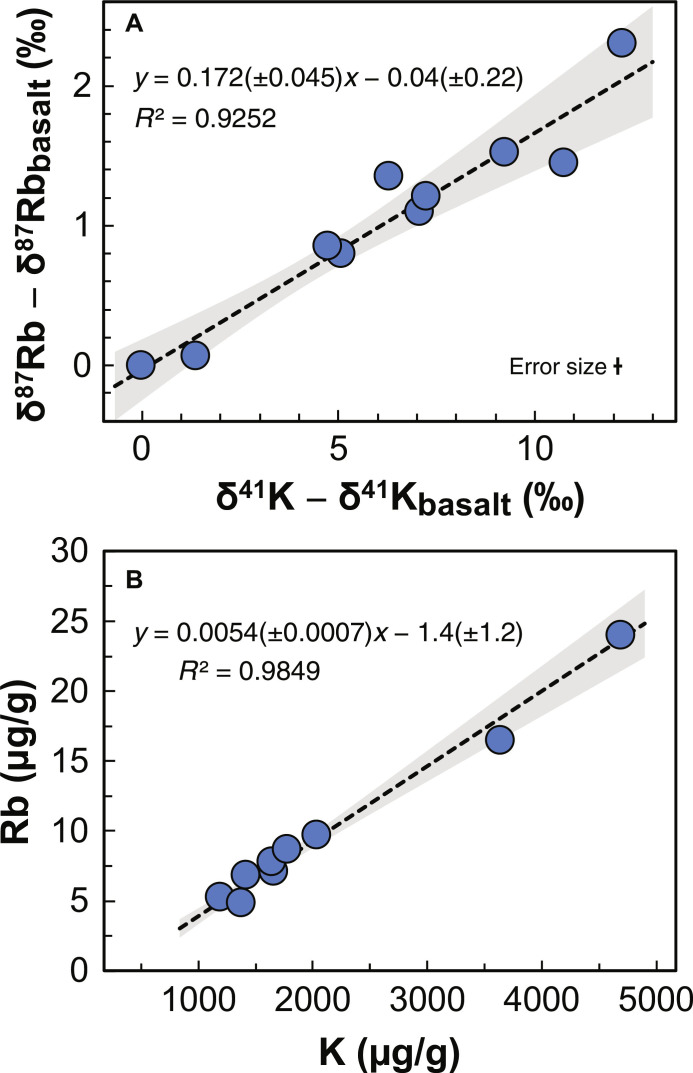
Potassium and rubidium isotopic and elemental correlations in lunar soil samples. (**A**) The isotopic compositions of K and Rb are linearly correlated with a slope of θ = 0.172 ± 0.045 (±95% CI). The isotopic compositions of K and Rb in the lunar basalt sample (δ^41^K_basalt_ = −0.22 ± 0.08‰ and δ^87^Rb_basalt_ = −0.09 ± 0.05‰, respectively; data S1A) were subtracted from those of lunar samples before conducting the linear regression. The heavy K and Rb isotopic compositions of lunar soils compared to lunar basalts reflect preferential loss of light isotopes to space. (**B**) Elemental correlation between K and Rb. The variations in elemental concentrations are caused by magmatic processes (see text for details). Blue circles are lunar samples, and the black dash lines and gray envelopes are linear fits of the data and the 95% CI uncertainties, respectively.

The K and Rb isotopic compositions are summarized in data S1A. The new K isotopic analyses agree with previous data (*13*, *18*–*20*) but with improved precision (fig. S1 and data S1B). The new Rb data reveal distinct Rb isotopic variations among the samples (Fig. 2 and fig. S1). The basalt has K and Rb isotopic compositions close to the bulk silicate Moon [both are close to zero (*13*–*17*)]. The lunar soils are enriched in heavy isotopes with large variations, ranging from +1.2 to +12‰ for K and −0.02 to +2.2‰ for Rb. The isotopic compositions of K and Rb are tightly correlated with a slope θ = δ^87^Rb/δ^41^K of 0.172 ± 0.045 (Fig. 2).

## DISCUSSION

### Isotopic fingerprints of atmosphere-soil interactions

The observed isotopic variations cannot be attributed to lunar magmatic differentiation, because they are an order of magnitude larger than those documented in lunar igneous rocks [variations of <1‰ for K and <0.2‰ for Rb in igneous rocks (*13*–*17*)]. Instead, the isotopic variations in lunar soils must reflect volatile loss caused by space weathering of lunar regolith over billions of years. Soils with more fractionated (heavier) isotopic compositions must have undergone prolonged exposure to space environments and/or more intense weathering. Plotting δ^41^K against Is/FeO, an index of soil maturity that measures the amount of fine-grained Fe metal (characterized by the intensity of ferromagnetic resonance Is) relative to FeO (*22*), reveals a broad trend between the two (fig. S2), consistent with space weathering influencing both quantities.

In contrast, no trend was observed between isotopic composition and elemental concentration (fig. S3), despite the tight correlation observed between K and Rb concentrations (Fig. 2), suggesting that different processes control elemental and isotopic fractionations. The large variations in K and Rb concentrations in the lunar soils correlate with variations in incompatible refractory elements like U and Ba (Supplement Materials and fig. S3). This shows that K and Rb concentration variations result from magmatic differentiation (*23*), which obscures the subtle elemental depletions associated with volatilization by space weathering. Therefore, elemental concentrations in lunar soils cannot be used to study atmosphere-soil interactions. While lunar surface weathering did not modify much the elemental concentrations, it left a large imprint on the isotopic composition of lunar soils, which can be used to trace the origin of the atoms in the lunar atmosphere.

### Different atmospheric sources and sinks impact isotopic compositions differently

The observed K-Rb isotopic variations and correlation result from long-term interactions between lunar regolith, atmosphere, and outer space through space weathering processes. Once primary atoms are liberated from the lunar surface by either IV or IS, a fraction of them undergo gravitational escape and are directly lost to space. The remaining atoms can bounce and be recycled between the regolith and the atmosphere before eventually being lost to space or returned to the lunar surface through photoionization and surface trapping (Fig. 1; see the Supplementary Materials for mathematical considerations). Among the three releasing mechanisms (IV, IS, and PSD), PSD plays a key role in recycling atmospheric atoms that return to the lunar surface at the end of a ballistic trajectory, where those atoms are weakly adsorbed (*24*–*29*). However, PSD is not thought to play a major role in the release of primary mineral-bound atoms (*10*, *30*). A major open question is then what the roles of IV and IS are in releasing surface atoms to sustain the lunar atmosphere (*6*, *7*, *31*–*33*).

Different source (IV and IS) and sink (gravitational escape associated with IV or IS, photoionization, and surface trapping) mechanisms for the lunar atmosphere, and combinations thereof, would produce distinct slopes (θ) for the δ^41^K-δ^87^Rb isotopic correlation in lunar soils, which can be compared with the observed slope of θ = 0.172 ± 0.045 to evaluate their contributions. We have calculated the predicted K-Rb isotopic slopes for different combinations of sources and sinks (see derivations in the Supplementary Materials). Below, we first discuss how the source and sink processes impact the K and Rb isotopic fractionations and then compare the observed isotopic slope θ in lunar soils to the predicted slopes for different endmember source-sink scenarios.

As atmospheric sources, both IV and IS preferentially liberate lighter isotopes from the lunar surface (*34*–*36*), thereby enriching the lunar atmosphere in lighter isotopes of K and Rb compared to the bulk lunar regolith, but by different degrees. For IV, experimental studies of K and Rb evaporation from silicate melts (*37*–*39*) suggest that the two elements behave very similarly during vaporization, meaning that the K/Rb ratio in the vapor should be close to the original value. The vaporized atoms are however isotopically fractionated relative to the source by about −22‰ for K and −10‰ for Rb assuming kinetic evaporation into vacuum (Supplementary Materials and data S1C). In comparison, IS liberates more K than Rb, with a Rb yield of about 30% lower than that of K (*40*), and the released atoms are predicted to be isotopically lighter relative to the source by about −5.5‰ for K and −2.6‰ for Rb (Supplementary Materials and data S1C) (*41*, *42*).

Both IV and IS are also energetic enough that a fraction of the liberated atoms gain speeds exceeding lunar escape velocity and are thus lost to space (*30*). The lost fractions and isotopic effects of gravitational escape associated with IV and IS can be calculated based on the energy distributions of the released atoms (*30*, *43*, *44*) (Supplementary Materials). Gravitational escape associated with IV is controlled by the temperature of the impacted materials (Supplementary Materials and fig. S4). Assuming a characteristic temperature of 4000 K (*30*, *43*), approximately 8% of K and 0.2% of Rb released by IV will escape gravity, with calculated isotopic fractionations between the escaping atoms and the bulk vapor of about −139‰ for K and −147‰ for Rb (data S1C; see data S1D and the Supplementary Materials for isotopic fractionations at other temperatures). Gravitational escape associated with IS is influenced by the mass and surface binding energy of the sputtered atoms. The atoms released by IS have higher energies and speeds (fig. S5) than IV, leading to higher escaped fractions of the sputtered atoms of ~88% for K and ~65% for Rb. The isotopic fractionations between the escaped atoms and the bulk sputtered atoms are approximately −10‰ for K and −12‰ for Rb (Supplementary Materials and data S1C).

The above calculations suggest that the processes of transferring atoms from the lunar surface to the atmosphere by IV and IS, together with the associated gravitational escape to space for a fraction of those atoms, can fractionate the isotopic compositions of lunar atmosphere and soils. The isotopic fractionations will differ depending on whether the source process is IV or IS.

**Fig. 3.**
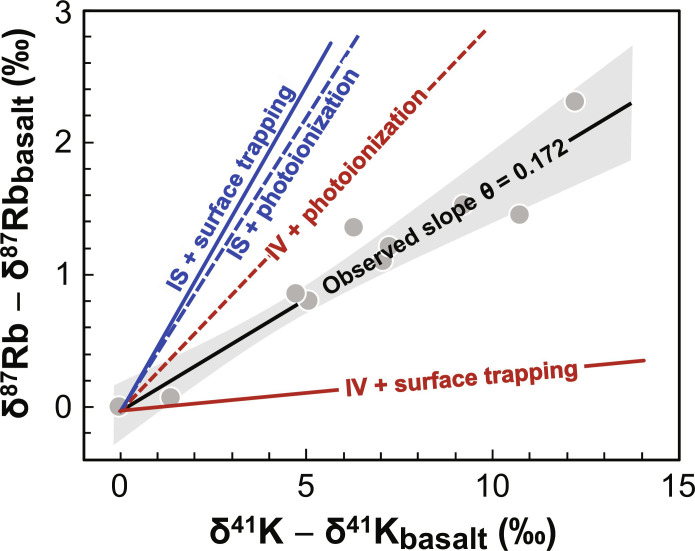
Comparison between observed K-Rb isotopic slope (θ = 0.172) of lunar soils and theoretical slopes for the four endmember atmospheric source-sink pairs. The endmember atmospheric source-sink pairs include: meteorite impact vaporization (IV) as the source and surface trapping as the sink (θ = 0.027), IV as the source and photoionization as the sink (θ = 0.293), ion sputtering (IS) as the source and photoionization as the sink (θ = 0.443), and IS as the source and surface trapping as the sink (θ = 0.493). The observed slope falls in between the four hypothetical slopes (red and blue lines), suggesting that a combination of those processes shaped the isotopic compositions of lunar soils. The gray circles show the measured K and Rb isotopic compositions of lunar soils, and the black line and gray area show the slope determined through linear regression and its associated uncertainty envelope, respectively.

The atmospheric sinks photoionization and surface trapping also affect how isotopes are fractionated in lunar soils, although the processes themselves are not expected to fractionate much the K/Rb ratio or their isotopic compositions (see discussions in the Supplementary Materials). Upon release by IV or IS, some atoms are directly lost to space through gravitational escape, and the remaining atoms linger in the atmosphere until they are either photoionized or trapped on the lunar surface. For those that are photoionized, they can either be lost to space (picked up by solar wind) or reimplanted onto the lunar surface (Fig. 1), with about equal chances (*45*). The reason why photoionization and surface trapping affect the isotopic composition of lunar soils is because they collectively determine the fraction of atmospheric atoms (those remaining after gravitational escape) that are lost to space or returned permanently into the regolith. If the atmospheric atoms are lost solely by surface trapping, then they will be returned to the soil and the main control of isotopic fractionation of lunar soils will be gravitational escape to space associated with IV and IS. The corresponding predicted slopes of the δ^41^K-δ^87^Rb correlation are θ = 0.027 if IV is the only source and 0.493 if IS is the only source (Fig. 3. The analytical equations for calculating those slopes are given by eqs. S43 and S44 in the Supplementary Materials). On the other hand, if photoionization is the dominant loss mechanism, then only approximately half of the atmospheric atoms will be returned to the soil (the rest will be lost to space), and isotopic fractionation in soils will be more affected by the release mechanisms IV and IS (in addition to isotope fractionation caused by gravitational escape). In this case, θ = 0.293 is expected if IV is the only source and θ = 0.443 if IS is the only source (Fig. 3).

Figure 3 compares the observed K-Rb isotopic slope in lunar soils (θ = 0.172 ± 0.045) with the slopes expected for the four-endmember source-sink pairs, considering two options for the dominant atmospheric sources (IV and IS) and two for the dominant sinks (photoionization and surface trapping; gravitational escape is tied to the release of atoms from the lunar surface by IV and IS and is not an independent loss mechanism). The observed slope falls in between the calculated slopes, indicating that a single source-sink pair cannot account for the signal seen in lunar soils. This is consistent with previous observations that the lunar atmosphere alkali contents respond to both meteor showers and lunar eclipses (the entry and exit of the Moon into and out of Earth’s magnetotail, where the Moon is shielded from sputtering by solar wind ions), implying both IV and IS as possible sources for the lunar atmosphere (*5*, *9*, *46*).

### Contributions of impact vaporization and ion sputtering to the lunar atmosphere

We have developed a mathematical model to constrain the contribution of each source and sink in sustaining the lunar atmosphere (Supplementary Materials and eqs. S19 to S42). The model calculates the K-Rb isotopic slope θ of lunar soils at steady state depending on the flux of atoms (ϕ) associated with each source and sink (ϕ_IV_, ϕ_IS_, ϕ_i_, and ϕ_tr_ denote the K atom fluxes associated with IV, IS, photoionization, and surface trapping, respectively). We found that two main parameters control the slope θ: the relative source fluxes of IS and IV ϕ_IS_/(ϕ_IS_ + ϕ_IV_), and the relative sink fluxes of photoionization and surface trapping ϕ_i_/(ϕ_i_ + ϕ_tr_) (eq. S41 in the Supplementary Materials).

**Fig. 4.**
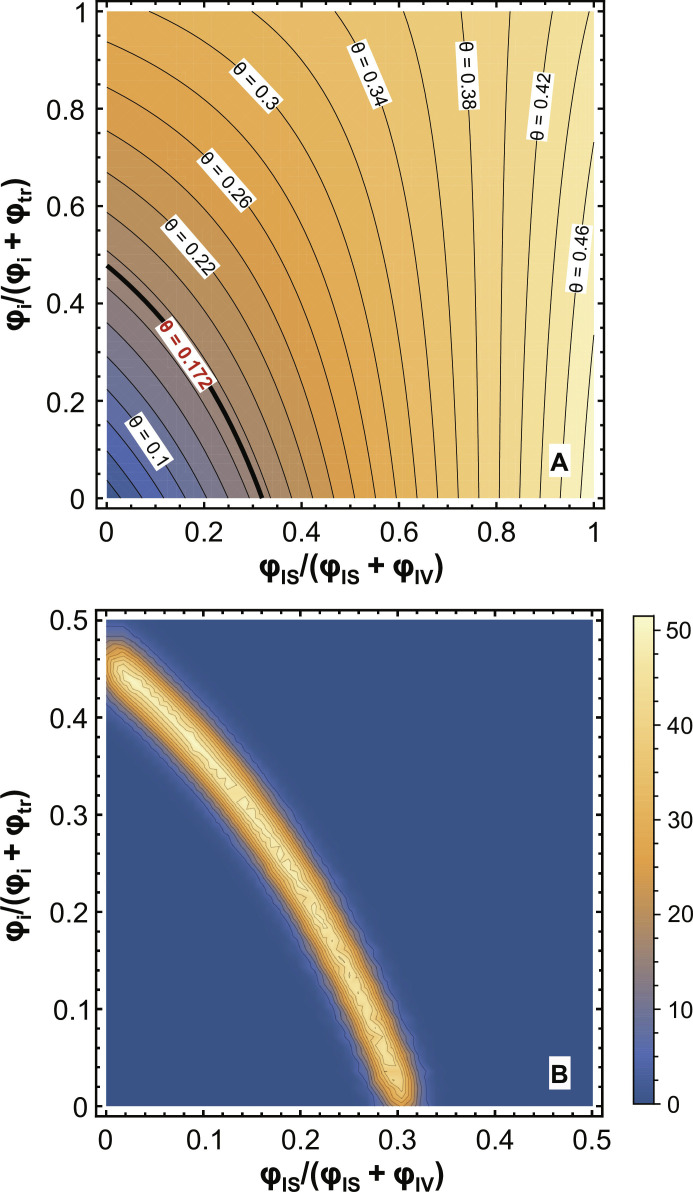
Relative contributions of lunar atmosphere sources and sinks constrained by the observed K-Rb isotopic slope (θ = 0.172). (**A**) K-Rb isotopic slope θ plotted as a function of two parameters, ϕ_IS_/(ϕ_IS_ + ϕ_IV_) and ϕ_i_/(ϕ_i_ + ϕ_tr_). ϕ_IS_/(ϕ_IS_ + ϕ_IV_) represents the flux of atoms released by ion sputtering (IS) divided by the total flux from meteorite impact vaporization (IV) and IS. ϕ_i_/(ϕ_i_ + ϕ_tr_) represents the flux of atmospheric atoms that are lost by photoionization (about half of photoionized atoms are lost to space and half reimplanted on the lunar surface) divided by the total flux lost through photoionization and surface trapping. The observed slope of 0.172 requires ϕ_IS_/(ϕ_IS_ + ϕ_IV_) to be between 0 and 0.32 and ϕ_i_/(ϕ_i_ + ϕ_tr_) between 0 and 0.48. This suggests that the predominate source of lunar atmosphere is meteoroid impact vaporization and the predominate sink is surface trapping. (**B**) The uncertainties of the two parameters calculated using a Markov Chain Monte Carlo (MCMC) method, shown as joint probability density distribution. Blue color indicates low posterior probability while yellow color indicates high posterior probability.

In Fig. 4, we show a contour plot of the predicted K-Rb isotopic slope θ of lunar soils as a function of the two unknowns ϕ_IS_/(ϕ_IS_ + ϕ_IV_) and ϕ_i_/(ϕ_i_ + ϕ_tr_) (both are bound between 0 and 1). The observed slope in lunar soils (θ = 0.172) constrains the two parameters to sit in narrow ranges. We find that ϕ_IS_/(ϕ_IS_ + ϕ_IV_) must be between 0 and 0.32 (Fig. 4A), indicating that IV dominates over IS as the major atmospheric source. We also find that ϕ_i_/(ϕ_i_ + ϕ_tr_) must be between 0 and 0.48 (Fig. 4A), indicating that surface trapping dominates over photoionization as the major sink for atoms that linger in the atmosphere (those that are not immediately lost by gravitational escape). The uncertainties of the two parameters were constrained using a Markov Chain Monte Carlo (MCMC) method (Supplementary Materials), and the probability density is shown in Fig. 4B. The two parameters are tightly correlated with a narrow range of uncertainties.

The calculated range for ϕ_IS_/(ϕ_IS_ + ϕ_IV_) is robust as it is not affected much by changing the values of the parameters used in the modeling (data S1C). Two important uncertainties are associated with IV: (i) the degree of vaporization of atoms from the lunar soil regolith into the lunar atmosphere and (ii) the temperature of atoms released by micrometeorite impacts (*47*).

During impact vaporization, kinetic effects impart strong isotopic fractionation to the original vapor, but as evaporation proceeds and more atoms are transferred to the vapor, its isotopic composition evolves toward the initial regolith composition. In the calculation above (Fig. 4), we assumed minimal vaporization, meaning that the isotopic fractionation between the vapor and the bulk regolith during impact vaporization is the largest conceivable. However, even if we assume 99.99% vaporization corresponding to little isotopic fractionation between vapor and bulk regolith, the calculated value of ϕ_IS_/(ϕ_IS_ + ϕ_IV_) is between 0.25 and 0.29, still consistent with impact vaporization representing the largest source of atoms in the lunar atmosphere (figs. S6 and S7). We however find that the value of ϕ_i_/(ϕ_i_ + ϕ_tr_) can now take values between 0 and 1, meaning that we cannot provide reliable constraints on sink mechanisms from the K and Rb isotopic data.

The temperature of released atoms controls the amount of gravitational escape associated with IV (fig. S4). A higher temperature leads to more gravitational escape and less K and Rb isotopic fractionations between the escaped and the impact-released atoms. A characteristic temperature of 4000 K was assumed in the above calculation, but the temperature could range from 2000 to 6000 K depending on the impact velocity (*48*, *49*). We have calculated the values of ϕ_IS_/(ϕ_IS_ + ϕ_IV_) and ϕ_i_/(ϕ_i_ + ϕ_tr_) at various temperatures ranging from 2000 to 6000 K (Supplementary Materials). The results show that ϕ_IS_/(ϕ_IS_ + ϕ_IV_) is always between 0 and 0.35 (figs. S8 and S9), again re-emphasizing the robustness of this estimate. The value of ϕ_i_/(ϕ_i_ + ϕ_tr_) is not sensitive to temperature change but varies with the assumed degree of impact vaporization, as discussed above, and is between 0 and 0.5 for minimal vaporization but can range from 0 to 1 for high degree of vaporization (figs. S8 and S9).

In summary, the observed K-Rb isotopic slope of lunar soils (θ = 0.172) indicates that impact vaporization is the dominant long-term source of the lunar atmosphere, likely contributing more than 65% of atmospheric K atoms, with ion sputtering accounting for the rest. Meteoroid impacts must have also vaporized other volatile elements, consistent with the observation that lunar soils are enriched in heavier isotopes of various volatile elements such as Si, Fe, Cu, Zn, and Cd (*34*, *50*–*54*). The constrained relative sink flux of photoionization and surface trapping ϕ_i_/(ϕ_i_ + ϕ_tr_) can vary from 0 to 1, depending on the assumed extent of vaporization during IV (figs. S6 to S9). Under our preferred assumption where the degree of impact vaporization is minimal, surface trapping appears to be the dominant sink of atmospheric atoms. This is consistent with modeling of lunar surface adsorption/desorption processes suggesting that surface trapping and not photoionization should be the major sink for Na in lunar atmosphere (*55*, *56*). However, further work is required to better understand the specifics of micrometeorite impact processes, notably the degree of vaporization and the temperature of the impacted material, as these factors influence the conclusions about the nature of the atmospheric sinks.

The lunar soil record provides quantitative insights into the long-term evolution of the lunar atmosphere that is not accessible with direct observations. Planetary regolith of more distant objects that have been or are currently targeted by sample return missions such as martian moon Phobos may have experienced similar volatile loss as is seen on the Moon. Measuring K and Rb isotopes in the regolith of those objects will help us understand how they were affected by meteoroid bombardments and solar wind sputtering on geological timescales and how space weathering differs across the solar system.

## MATERIALS AND METHODS

### Sample digestion and column chromatography for K and Rb purification

The lunar soil samples were provided by NASA. The samples were digested using HF, HNO_3_, and HCl following a protocol that has been previously applied to lunar and chondrite samples (*14*, *57*, *58*). Approximately 20 to 60 mg of each homogenized sample powder, with original mass exceeding 100 mg, was placed in Savillex perfluoroalkoxy (PFA) beakers and heated to 130°C in a concentrated HF + HNO_3_ solution (2:1 volume mixture of 28 M HF and 15 M HNO_3_) for 24 hours. The resulting sample solutions were dried and redissolved twice in aqua regia (3:1 volume mixture of 11 M HCl and 15 M HNO_3_) at 130°C for 24 hours each. Subsequently, the solutions were dried, redissolved in 1 M HNO_3_ at 130°C for 24 hours, dried again, and lastly redissolved in 1 M HNO_3_ for ion exchange column chromatography.

The column chromatography procedure allows simultaneously purification of K and Rb from the same digested samples (*14*, *21*). Quantitatively retrieving purified K and Rb from the same digested sample is vital for establishing a precise K-Rb isotopic correlation. However, this is usually difficult to achieve due to the very different concentrations of the two elements (the K/Rb ratios in the samples are around 200) and their similar behaviors during chromatography. The method used here consisted of four columns to remove matrix elements and progressively concentrate and separate K and Rb. In the first step, sample solutions in 1 M HNO_3_ were loaded onto Bio-Rad Econo-Pac columns containing 16-ml AG50W-X8 resin (200 to 400 mesh). Each column was flushed with 160 ml of 1 M HNO_3_ to collect alkali metal elements. After drying and redissolving in 0.5 M HNO_3_, the solutions underwent a second round of separation on the same resin columns (cleaned with 240 ml of 6 M HNO_3_). Matrix elements were eluted with 130 ml of 0.5 M HNO_3_, followed by elution of Rb and K with 300 ml of 0.5 M HNO_3_. The K-Rb fraction was further purified by removing Ti using a 1-ml column of AG1-X8 anion exchange resin (200 to 400 mesh). Rubidium and K were directly eluted from the column with 9 ml of 2 M HF, while Ti remained bound to the resin. The solutions were then evaporated and dissolved in 3 M HNO_3_ for the final chromatography step using Savillex PFA microcolumns (0.45 cm internal diameter and 40 cm length) filled with Eichrom crown ether extractant Sr resin (50 to 100 μm). Sample solutions in 0.1 ml of 3 M HNO_3_ were loaded onto the Sr resin columns, followed by elution of matrix elements with 3.9 ml of 3 M HNO_3_ and subsequent collection of Rb using 12 ml of 3 M HNO_3_ and K using 20 ml of 3 M HNO_3_. The Sr resin step helps remove any remaining Sr, which could interfere with ^87^Rb^+^ signal during mass spectrometry. The overall yields of Rb and K from the procedure exceed 95%. Procedural blanks for Rb and K are <0.2 and <20 ng, respectively.

### Rubidium isotopic analyses

Rubidium isotopic analyses were conducted at the Origins Lab of the University of Chicago using a Thermo Fisher Scientific Neptune Plus MC-ICPMS, following an established protocol (*14*, *21*). Purified Rb solutions in 2% HNO_3_ were introduced into the torch using a 100 μl/min PFA nebulizer and dual Scott-cyclonic quartz spray chamber (stable introduction system), measured with standard nickel sampler and H skimmer cones at low resolution. Rubidium isotopes were measured at a signal intensity of 1 to 2 V with concentrations of 15 to 30 ng/g of Rb. The signal at ^88^Sr^+^ was monitored for correcting for ^87^Sr^+^ interference on ^87^Rb^+^, assuming an ^87^Sr^+^/^88^Sr^+^ ratio of 0.085. Each sample analysis consisted of a single block of 25 cycles, with an integration time of 4.194 s per cycle. Instrumental fractionation was corrected for using the standard-sample bracketing method. Acid blanks were measured before and after analyzing each standard or sample, and the average was subtracted from the signal intensity of the bracketed standard or sample. Each purified sample solution was measured 7 to 10 times, and the average value was taken as the sample’s isotopic composition. The uncertainty of the sample was calculated as the 95% CI using the formula 2σ / √ *n*, where *n* represents the number of replicate analyses of the sample, and σ the SD of the standards bracketed by itself during each analytical session. The σ value of the standard was used because it was analyzed many more times than each sample during a session and provides a better measure of instrumental stability. Rubidium isotopic compositions were expressed using the δ-notation against the reference material NIST SRM984.

### Potassium isotopic analyses

Potassium isotopic analyses of the lunar soil samples using Neptune Plus were carried out at the Origins Lab at the University of Chicago using a “cold plasma” method (*59*, *60*). Potassium solutions in 2% HNO_3_ were introduced into the torch using a 100 μl/min PFA nebulizer and an Apex Omega high sensitivity desolvation system and measured with standard nickel sampler and H skimmer cones at high mass resolution. Potassium isotopes were measured at a signal intensity of 8 to 12 V for ^39^K, at a K concentration of ~1 µg/g, which is comparable to previous studies using the same type of instrument (*59*).The radio frequency power was reduced to 650 W from the normal setting of 1300 W to reduce argide interferences, in particular ^40^Ar^1^H^+^ on ^41^K^+^. The cup configuration followed previous studies (*59*). Instrumental fractionation was corrected for using the standard-sample bracketing method. Acid blanks were measured before and after blocks of four sample-standard analyses, and the average was subtracted from the measured signal intensities of the standards and samples in between. Each sample was measured eight to nine times, and the average value was taken as the isotopic composition of the sample. The uncertainty of the sample was calculated as the 95% CI using the same formula as given for Rb. Potassium isotopic compositions were reported using the δ-notation against the reference material NIST SRM999c, which has the same K isotopic composition as the reference material SRM 3141a (*60*).

Potassium isotopic analysis by conventional MC-ICPMS is complicated by the presence of potential isobaric and molecular interferences from the argon gas used for the plasma, notably ^38^ArH^+^ on ^39^K^+^, ^40^Ar^+^ on ^40^K^+^, and ^40^ArH^+^ on ^41^K^+^. We also conducted an initial test of the potential of K isotopic analyses with a Thermo Fisher Scientific Neoma collision cell (CC)-MC-ICP-MS/MS. We measured the K isotopic compositions of both geostandards and lunar soil samples using the Neoma CC-MC-ICP-MS/MS, and consistent results were obtained, with precisions comparable to the Nu Instruments Sapphire CC-MC-ICP-MS (see the Supplementary Materials for details). The data are summarized in data S1 (E to G) and the Supplementary Materials.

## Supplementary Materials

### This PDF file includes:

Supplementary Text Figs. S1 to S10 Legend for data S1 References

## References

[1] D. M. Hunten, A. L. Sprague, Origin and character of the lunar and mercurian atmospheres. Adv. Space Res. 19, 1551–1560 (1997).

[2] R. M. Killen, W.-H. Ip, The surface-bounded atmospheres of Mercury and the Moon. Rev. Geophys. 37, 361–406 (1999).

[3] A. Potter, T. Morgan, Discovery of sodium and potassium vapor in the atmosphere of the Moon. Science 241, 675–680 (1988). 17839078 10.1126/science.241.4866.675

[4] A. L. Tyler, R. W. Kozlowski, D. M. Hunten, Observations of sodium in the tenuous lunar atmosphere. Geophys. Res. Lett. 15, 1141–1144 (1988).

[5] S. M. Smith, J. K. Wilson, J. Baumgardner, M. Mendillo, Discovery of the distant lunar sodium tail and its enhancement following the Leonid Meteor Shower of 1998. Geophys. Res. Lett. 26, 1649–1652 (1999).

[6] S. A. Stern, The lunar atmosphere: History, status, current problems, and context. Rev. Geophys. 37, 453–491 (1999).

[7] W. H. Smyth, M. L. Marconi, Theoretical overview and modeling of the sodium and potassium atmospheres of the Moon. Astrophys. J. 443, 371–392 (1995).

[8] J. R. Szalay, M. Horányi, A. Colaprete, M. Sarantos, Meteoritic influence on sodium and potassium abundance in the lunar exosphere measured by LADEE. Geophys. Res. Lett. 43, 6096–6102 (2016).

[9] A. Colaprete, M. Sarantos, D. H. Wooden, T. J. Stubbs, A. M. Cook, M. Shirley, How surface composition and meteoroid impacts mediate sodium and potassium in the lunar exosphere. Science 351, 249–252 (2016). 26678876 10.1126/science.aad2380

[10] A. L. Sprague, R. W. H. Kozlowski, D. M. Hunten, W. K. Wells, F. A. Grosse, The sodium and potassium atmosphere of the moon and its interaction with the surface. Icarus 96, 27–42 (1992).

[11] R. M. Killen, T. H. Morgan, A. E. Potter, G. Bacon, I. Ajang, A. R. Poppe, Coronagraphic observations of the lunar sodium exosphere 2018–2019. Icarus 355, 114155 (2021).

[12] B. Fegley Jr., K. Lodders, N. S. Jacobson, Chemical equilibrium calculations for bulk silicate earth material at high temperatures. Geochemistry 83, 125961 (2023).

[13] K. Wang, S. B. Jacobsen, Potassium isotopic evidence for a high-energy giant impact origin of the Moon. Nature 538, 487–490 (2016). 27617635 10.1038/nature19341

[14] N. X. Nie, N. Dauphas, Vapor drainage in the protolunar disk as the cause for the depletion in volatile elements of the Moon. Astrophys. J. 884, L48 (2019).

[15] E. A. Pringle, F. Moynier, Rubidium isotopic composition of the Earth, meteorites, and the Moon: Evidence for the origin of volatile loss during planetary accretion. Earth Planet. Sci. Lett. 473, 62–70 (2017).

[16] N. X. Nie, X. Y. Chen, Z. J. Zhang, J. Y. Hu, W. Liu, F. L. H. Tissot, F. Z. Teng, A. Shahar, N. Dauphas, Rubidium and potassium isotopic variations in chondrites and Mars: Accretion signatures and planetary overprints. Geochim. Cosmochim. Acta 344, 207–229 (2023).

[17] Z. Tian, B. L. Jolliff, R. L. Korotev, B. Fegley Jr., K. Lodders, J. M. D. Day, H. Chen, K. Wang, Potassium isotopic composition of the Moon. Geochim. Cosmochim. Acta 280, 263–280 (2020).

[18] E. L. Garner, L. A. Machlan, I. L. Barnes, The isotopic composition of lithium, potassium, and rubidium in some Apollo 11, 12, 14, 15, and 16 samples, in *Proceedings of the Sixth Lunar Science Conference* (Houston, Texas, 17 to 21 March 1975), pp. 1845–1855.

[19] S. E. Church, G. R. Tilton, J. E. Wright, C.-N. Lee-Hu, Volatile element depletion and K-39/K-41 fractionation in lunar soils, in *Proceeding of the Seventh Lunar Science Conference* (Houston, Texas, 15 to 19 March 1976), pp. 423–439.

[20] M. Humayun, R. N. Clayton, Precise determination of the isotopic composition of potassium: Application to terrestrial rocks and lunar soils. Geochim. Cosmochim. Acta 59, 2115–2130 (1995).

[21] N. X. Nie, N. Dauphas, T. Hopp, J. Y. Hu, Z. J. Zhang, R. Yokochi, T. J. Ireland, F. L. H. Tissot, Chromatography purification of Rb for accurate isotopic analysis by MC-ICPMS: A comparison between AMP-PAN, cation-exchange, and Sr resins. J. Anal. At. Spectrom. 36, 2588–2602 (2021).

[22] R. V. Morris, Surface exposure indices of lunar soils-A comparative FMR study, in *Proceedings of the Seventh Lunar Science Conference, Houston, Texas, 15 to 19 March 1976* (Pergamon Press Inc., New York, 1976), vol. 1, pp. 315–335.

[23] N. Dauphas, N. X. Nie, M. Blanchard, Z. J. Zhang, H. Zeng, J. Y. Hu, M. Meheut, C. Visscher, R. Canup, T. Hopp, The extent, nature, and origin of K and Rb depletions and isotopic fractionations in Earth, the Moon, and other planetary bodies. Planet. Sci. J. 3, 29 (2022).

[24] B. V. Yakshinskiy, T. E. Madey, Photon-stimulated desorption as a substantial source of sodium in the lunar atmosphere. Nature 400, 642–644 (1999). 10458159 10.1038/23204

[25] B. V. Yakshinskiy, T. E. Madey, Desorption induced by electronic transitions of Na from SiO2: Relevance to tenuous planetary atmospheres. Surf. Sci. 451, 160–165 (2000).

[26] B. V. Yakshinskiy, T. E. Madey, Electron- and photon-stimulated desorption of K from ice surfaces. J. Geophys. Res. Planets 106, 33303–33307 (2001).

[27] B. V. Yakshinskiy, T. E. Madey, Photon-stimulated desorption of Na from a lunar sample: Temperature-dependent effects. Icarus 168, 53–59 (2004).

[28] M. Sarantos, R. M. Killen, A. Surjalal Sharma, J. A. Slavin, Sources of sodium in the lunar exosphere: Modeling using ground-based observations of sodium emission and spacecraft data of the plasma. Icarus 205, 364–374 (2010).

[29] M. Kagitani, M. Taguchi, A. Yamazaki, I. Yoshikawa, G. Murakami, K. Yoshioka, S. Kameda, S. Okano, Variation in lunar sodium exosphere measured from lunar orbiter SELENE (Kaguya). Planet. Space Sci. 58, 1660–1664 (2010).

[30] P. Wurz, S. Fatemi, A. Galli, J. Halekas, Y. Harada, N. Jäggi, J. Jasinski, H. Lammer, S. Lindsay, M. N. Nishino, T. M. Orlando, J. M. Raines, M. Scherf, J. Slavin, A. Vorburger, R. Winslow, Particles and photons as drivers for particle release from the surfaces of the Moon and Mercury. Space Sci. Rev. 218, 10 (2022).

[31] P. K. Haff, Z. E. Switkowski, D. S. Burnett, T. A. Tombrello, Gravitational and recoil contributions to surface mass fractionation by solar-wind sputtering, in *Proceeding of the 8th Lsunar Science Conference, Houston, Texas, 14 to 18March 1977* (Pergamon Press Inc., New York, 1977), pp. 3807–3815.

[32] R. M. Housley, A model for chemical and isotopic fractionation in the lunar regolith by impact vaporization, in *Proceedings of the 10th Lunar and Planetary Science Conference, Houston, Texas, 19 to 23 March 1979* (Pergamon, Press Inc., 1979), vol. 2, pp. 1673–1683.

[33] T. H. Morgan, H. A. Zook, A. E. Potter, Production of sodium vapor from exposed regolith in the inner solar system, in *Proceedingas of the 19th Lunar and Planetary Science Conference, Houston, Texas, 14 to 18 March 1988,* (Cambridge/Houston, Texas, Cambridge Univ. Press/Lunar and Planetary Institute, 1989), pp. 297–304.

[34] R. Clayton, T. Mayeda, J. Hurd, Loss of oxygen, silicon, sulfur, and potassium from the lunar regolith, in *Proceedings of the 5th Lunar and Planetary Science Conference Houston, Texas, 18 to 22 March 1974* (Pergamon Press Inc., New York, 1974), vol. 2, pp. 1801–1809.

[35] Z. L. Liau, W. L. Brown, R. Homer, J. M. Poate, Surface-layer composition changes in sputtered alloys and compounds. Appl. Phys. Lett. 30, 626–628 (1977).

[36] W. A. Russell, D. A. Papanastassiou, T. A. Tombrello, The fractionation of Ca isotopes by sputtering. Radiat. Eff. 52, 41–52 (1980).

[37] Y. Yu, R. H. Hewins, C. M. O. ’D. Alexander, J. Wang, Experimental study of evaporation and isotopic mass fractionation of potassium in silicate melts. Geochim. Cosmochim. Acta 67, 773–786 (2003).

[38] F. M. Richter, R. A. Mendybaev, J. N. Christensen, D. Ebel, A. Gaffney, Laboratory experiments bearing on the origin and evolution of olivine-rich chondrules. Meteorit. Planet. Sci. 46, 1152–1178 (2011).

[39] Z. J. Zhang, N. X. Nie, R. A. Mendybaev, M.-C. Liu, J. J. Hu, T. Hopp, E. E. Alp, B. Lavina, E. S. Bullock, K. D. McKeegan, Loss and isotopic fractionation of alkali elements during diffusion-limited evaporation from molten silicate: Theory and experiments. ACS Earth Space Chem. 5, 755–784 (2021).

[40] R. L. Hervig, F. K. Mazdab, P. Williams, Y. Guan, G. R. Huss, L. A. Leshin, Useful ion yields for Cameca IMS 3f and 6f SIMS: Limits on quantitative analysis. Chem. Geol. 227, 83–99 (2006).

[41] P. Sigmund, Theory of sputtering. I. Sputtering yield of amorphous and polycrystalline targets. Phys. Rev. 184, 383–416 (1969).

[42] P. Sigmund, Mechanisms and theory of physical sputtering by particle impact. Nucl. Instrum. Methods Phys. Res. B 27, 1–20 (1987).

[43] P. Wurz, J. A. Whitby, U. Rohner, J. A. Martín-Fernández, H. Lammer, C. Kolb, Self-consistent modelling of Mercury’s exosphere by sputtering, micro-meteorite impact and photon-stimulated desorption. Planet. Space Sci. 58, 1599–1616 (2010).

[44] D. Gamborino, P. Wurz, Velocity distribution function of Na released by photons from planetary surfaces. Planet. Space Sci. 159, 97–104 (2018).

[45] R. Manka, F. Michel, Lunar atmosphere as a source of argon-40 and other lunar surface elements. Science 169, 278–280 (1970). 17752540 10.1126/science.169.3942.278

[46] A. E. Potter, T. H. Morgan, Variation of lunar sodium emission intensity with phase angle. Geophys. Res. Lett. 21, 2263–2266 (1994).

[47] G. Herzog, F. Moynier, F. Albarède, A. Berezhnoy, Isotopic and elemental abundances of copper and zinc in lunar samples, Zagami, Pele’s hairs, and a terrestrial basalt. Geochim. Cosmochim. Acta 73, 5884–5904 (2009).

[48] G. Eichhorn, Impact light flash studies: Temperature, ejecta, vaporization, in *Interplanetary Dust and Zodiacal Light*. *Lecture Notes in Physics,* H. Elsässer, H. Fechting, Eds. (Springer, Berlin, Heidelberg, 1976), pp. 243–247.

[49] G. Eichhorn, Heating and vaporization during hypervelocity particle impact. Planet. Space Sci. 26, 463–467 (1978).

[50] S. Epstein, H. P. Taylor Jr., Investigation of the carbon, hydrogen, oxygen, and silicon isotope and concentration relationships on the grain surfaces of a variety of lunar soils and in some Apollo 15 and 16 core samples, in *Proceedings of the 6th Lunar Science Conference, Houston, Texas, 17 to 21 March 1975,* (Pergamon Press Inc., New York, 1975), vol. 2, pp. 1771–1798.

[51] D. G. Sands, K. J. R. Rosman, J. R. de Laeter, A preliminary study of cadmium mass fractionation in lunar soils. Earth Planet. Sci. Lett. 186, 103–111 (2001).

[52] R. A. Wiesli, B. L. Beard, L. A. Taylor, C. M. Johnson, Space weathering processes on airless bodies: Fe isotope fractionation in the lunar regolith. Earth Planet. Sci. Lett. 216, 457–465 (2003).

[53] F. Moynier, F. Albarède, G. F. Herzog, Isotopic composition of zinc, copper, and iron in lunar samples. Geochim. Cosmochim. Acta 70, 6103–6117 (2006).

[54] K. Wang, F. Moynier, F. A. Podosek, J. Foriel, An iron isotope perspective on the origin of the nanophase metallic iron in lunar regolith. Earth Planet. Sci. Lett. 337-338, 17–24 (2012).

[55] V. Tenishev, M. Rubin, O. J. Tucker, M. R. Combi, M. Sarantos, Kinetic modeling of sodium in the lunar exosphere. Icarus 226, 1538–1549 (2013).

[56] M. Sarantos, S. Tsavachidis, The boundary of alkali surface boundary exospheres of Mercury and the Moon. Geophys. Res. Lett. 47, e2020GL088930 (2020).

[57] N. X. Nie, X.-Y. Chen, T. Hopp, J. Y. Hu, Z. J. Zhang, F.-Z. Teng, A. Shahar, N. Dauphas, Imprint of chondrule formation on the K and Rb isotopic compositions of carbonaceous meteorites. Sci. Adv. 7, eabl3929 (2021). 34851657 10.1126/sciadv.abl3929 PMC8635422

[58] N. X. Nie, D. Wang, Z. A. Torrano, R. W. Carlson, C. M. O’D. Alexander, A. Shahar, Meteorites have inherited nucleosynthetic anomalies of potassium-40 produced in supernovae. Science 379, 372–376 (2023). 36701465 10.1126/science.abn1783

[59] H. Chen, Z. Tian, B. Tuller-Ross, R. L. Korotev, K. Wang, High-precision potassium isotopic analysis by MC-ICP-MS: An inter-laboratory comparison and refined K atomic weight. J. Anal. At. Spectrom 34, 160–171 (2019).

[60] Y. Hu, X.-Y. Chen, Y.-K. Xu, F.-Z. Teng, High-precision analysis of potassium isotopes by HR-MC-ICPMS. Chem. Geol. 493, 100–108 (2018).

[61] W. Li, B. L. Beard, S. Li, Precise measurement of stable potassium isotope ratios using a single focusing collision cell multi-collector ICP-MS. J. Anal. At. Spectrom 31, 1023–1029 (2016).

[62] W. Li, M. Cui, Q. Pan, J. Wang, B. Gao, S. Liu, M. Yuan, B. Su, Y. Zhao, F.-Z. Teng, High-precision potassium isotope analysis using the Nu Sapphire collision cell (CC)-MC-ICP-MS. Sci. China Earth Sci. 65, 1510–1521 (2022).

[63] K. Wang, S. B. Jacobsen, An estimate of the Bulk Silicate Earth potassium isotopic composition based on MC-ICPMS measurements of basalts. Geochim. Cosmochim. Acta 178, 223–232 (2016).

[64] Y. Ku, S. B. Jacobsen, Potassium isotope anomalies in meteorites inherited from the protosolar molecular cloud. Sci. Adv. 6, eabd0511 (2020). 33036981 10.1126/sciadv.abd0511 PMC7546711

[65] H. Chen, N. J. Saunders, M. Jerram, A. N. Halliday, High-precision potassium isotopic measurements by collision cell equipped MC-ICPMS. Chem. Geol. 578, 120281 (2021).

[66] F. Moynier, Y. Hu, K. Wang, Y. Zhao, Y. Gérard, Z. Deng, J. Moureau, W. Li, J. I. Simon, F.-Z. Teng, Potassium isotopic composition of various samples using a dual-path collision cell-capable multiple-collector inductively coupled plasma mass spectrometer Nu instruments Sapphire. Chem. Geol. 571, 120144 (2021).

[67] X.-Y. Zheng, X.-Y. Chen, W. Ding, Y. Zhang, S. Charin, Y. Gérard, High precision analysis of stable potassium (K) isotopes by the collision cell MC-ICP-MS “Sapphire” and a correction method for concentration mismatch. J. Anal. At. Spectrom 37, 1273–1287 (2022).

[68] P. Télouk, E. Albalat, T. Tacail, F. Arnaud-Godet, V. Balter, Steady analyses of potassium stable isotopes using a Thermo Scientific Neoma MC-ICP-MS. J. Anal. At. Spectrom 37, 1259–1264 (2022).

[69] G. Craig, H. Wehrs, D. G. Bevan, M. Pfeifer, J. Lewis, C. D. Coath, T. Elliott, C. Huang, N. S. Lloyd, J. B. Schwieters, Project vienna: A novel precell mass filter for collision/reaction cell MC-ICPMS/MS. Anal. Chem. 93, 10519–10527 (2021). 34282898 10.1021/acs.analchem.1c01475

[70] N. Dauphas, T. Hopp, G. Craig, Z. J. Zhang, M. C. Valdes, P. R. Heck, B. L. A. Charlier, E. A. Bell, T. Mark Harrison, A. M. Davis, L. Dussubieux, P. R. Williams, M. J. Krawczynski, C. Bouman, N. S. Lloyd, D. Tollstrup, J. B. Schwieters, In situ ^87^Rb–^87^Sr analyses of terrestrial and extraterrestrial samples by LA-MC-ICP-MS/MS with double Wien filter and collision cell technologies. J. Anal. At. Spectrom 37, 2420–2441 (2022).

[71] Y.-K. Xu, Y. Hu, X.-Y. Chen, T.-Y. Huang, R. S. Sletten, D. Zhu, F.-Z. Teng, Potassium isotopic compositions of international geological reference materials. Chem. Geol. 513, 101–107 (2019).

[72] F. M. Richter, Timescales determining the degree of kinetic isotope fractionation by evaporation and condensation. Geochim. Cosmochim. Acta 68, 4971–4992 (2004).

[73] N. Dauphas, F. Poitrasson, C. Burkhardt, H. Kobayashi, K. Kurosawa, Planetary and meteoritic Mg/Si and δ30Si variations inherited from solar nebula chemistry. Earth Planet. Sci. Lett. 427, 236–248 (2015).

[74] F. M. Richter, P. E. Janney, R. A. Mendybaev, A. M. Davis, M. Wadhwa, Elemental and isotopic fractionation of type B CAI-like liquids by evaporation. Geochim. Cosmochim. Acta 71, 5544–5564 (2007).

[75] F. M. Richter, A. M. Davis, D. S. Ebel, A. Hashimoto, Elemental and isotopic fractionation of type B calcium-, aluminum-rich inclusions: Experiments, theoretical considerations, and constraints on their thermal evolution. Geochim. Cosmochim. Acta 66, 521–540 (2002).

[76] G. De Maria, G. Balducci, M. Guido, V. Piacente, Mass spectrometric investigation of the vaporization process of Apollo 12 lunar samples. Proc. Lunar Sci. Conf. 2, 1367–1380 (1971).

[77] J. J. Naughton, J. V. Derby, V. A. Lewis, Vaporization from heated lunar samples and the investigation of lunar erosion by volatilized alkalis. Proc. Lunar Sci. Conf. 2, 449–457 (1971).

[78] E. K. Gibson Jr., N. J. Hubbard, Thermal volatilization studies on lunar samples. Proc. Lunar Sci. Conf. 3, 2003–2014 (1972).

[79] Y. Kudriavtsev, A. Villegas, A. Godines, R. Asomoza, Calculation of the surface binding energy for ion sputtered particles. Appl. Surf. Sci. 239, 273–278 (2005).

[80] P. Wurz, U. Rohner, J. A. Whitby, C. Kolb, H. Lammer, P. Dobnikar, J. A. Martín-Fernández, The lunar exosphere: The sputtering contribution. Icarus 191, 486–496 (2007).

[81] A. M. Davis, Volatile evolution and loss, in *Meteorites and the early solar system II* (University of Arizona Press, 2006), p. 295–307.

[82] R. V. Morris, The surface exposure/maturity/of lunar soils—Some concepts and I_s/FeO_ compilation, in *Proceedings of the 9th Lunas and Planetary Science Conference, Houston, Texas, 13 to 17 March 1978* (Pergamon Press Inc., 1978) vol. 2, pp. 2287–2297.

[83] T. Magna, U. Wiechert, A. N. Halliday, New constraints on the lithium isotope compositions of the Moon and terrestrial planets. Earth Planet. Sci. Lett. 243, 336–353 (2006).

[84] I. Barnes, E. Garner, J. Gramlich, L. Machlan, J. Moody, L. Moore, T. Murphy, W. Shields, Isotopic abundance ratios and concentrations of selected elements in some Apollo 15 and Apollo 16 samples. Proc. Lunar Sci. Conf., 4, 1197–1207 (1973).

