# Supplementary material for: Lunar soil record of atmosphere loss over eons
Source: Sci Adv. 2024 Aug 2;10(31):eadm7074. doi: 10.1126/sciadv.adm7074 (PMC11296337; doi:10.1126/sciadv.adm7074)
Supplement: Supplementary file 1 — Supplementary Text Figs. S1 to S10 Legend for data S1 References [file sciadv.adm7074_sm.pdf]

Science Advances
AAAS

Supplementary Materials for

Lunar soil record of atmosphere loss over eons

Nicole X. Nie *et al.*

Corresponding author: Nicole X. Nie, nxnie@mit.edu

Sci. Adv. **10**, eadm7074 (2024)
DOI: 10.1126/sciadv.adm7074

The PDF file includes:

Supplementary text
Figs. S1 to S10
Legend for data S1
References

Other Supplementary Material for thismanuscript includes the following:

Data S1

Supplementary Text

<u>Potassium isotopic measurements using Neoma CC-MC-ICP-MS/MS and dual loop syringe injection system</u>

Potassium isotopic analysis by conventional MC-ICP-MS such as Neptune Plus) is complicated by the presence of potential isobaric and molecular interferences from the argon gas used for the plasma ($^{38}ArH^+$ on $^{39}K^+$, $^{40}Ar^+$ on $^{40}K^+$, and $^{40}ArH^+$ on $^{41}K^+$). To resolve these interferences, samples are usually measured with high-resolution slits under "cold plasma" conditions, which require the use of more concentrated sample solutions because the method lowers the overall sensitivity of the instrument (*59*, *60*). MC-ICP-MS equipped with a collision cell can remove argide interferences through collision with H_2 gas, allowing K isotopic measurements in low mass resolution using conventional "hot plasma" (*38*, *61–67*). We decided to conduct a test of the potential of K isotopic analyses with a ThermoScientific Neoma-MS/MS. The extra-high resolution capability of this instrument has been used to measure K isotopes (*68*), but K isotopic measurements utilizing the collision cell capability of this instrument are rare.

The MS/MS component of Neoma comprises a pre-cell double Wien filter (pre-cell mass filter) and a collision/reaction cell (CRC). It can be tuned to only transmit ions within a specific mass range by adjusting the strength of the applied electric and magnetic fields of the Wien filters and the size of the aperture of the variable slit in between, and is very useful in removing argide interferences or react an element of interest to an interference free mass range (*e.g.*, $^{8x}Sr^+ + SF_6 \rightarrow {}^{8x}SrF^+$) (*69*, *70*). For K isotopic analysis, the pre-cell mass filter cannot be used to remove argide interferences because of the similar masses of Ar and K. Therefore, the pre-cell mass filter is set to a low magnetic field and a wide slit opening to operate in "transmission mode" that allows transmission of a large mass window (~20 to ~100 amu). Argide interferences on $^{39}K^+$ and $^{41}K^+$ are removed by charge exchange during collision with H_2 in the CRC. Additionally, He gas is used as a buffer gas in the CRC to improve transmission.

The K isotopic compositions of lunar soils were measured using a Neoma MS/MS in combination with an *ESI* Apex *Omega Q* and an *ESI* micro*FAST Isotope* dual loop syringe loading and injection system at the *ThermoFisher* factory in Bremen, Germany. The operational parameters of the setup are summarized in Data S1E. Potassium isotopic compositions were measured using standard-sample bracketing to correct for instrumental mass fractionation. The K reference standard SRM3141a was measured in 3% HNO_3 using a concentration of 100 ng/g and a flow rate of 50 µL/min. Sample solutions were measured using the same 3% HNO_3 solution but at variable K concentrations, and the signals of samples and the bracketing SRM3141a were matched by manually adjusting the flow rates individually using the *ESI* micro*FAST Isotope* dual loop syringe loading and injection system.

Potassium isotopic data were collected in static mode with $^{39}K^+$ in the center faraday cup (C) and $^{41}K^+$ in the H2 cup. Possible signals of $^{38}Ar^+$, $^{40}Ca^+$, $^{42}Ca^+$, $^{43}Ca^+$, and $^{44}Ca^+$ ions were monitored on L1, H1, H3, H4, and H5 cups. All faraday cups are connected to $10^{11}\Omega$ resistors. Before each standard and sample measurement, a wash of 3% HNO_3 for 5 min was performed and an on peak zero (OPZ) of 3% HNO_3 was measured. All measurements consisted of 75 cycles of 4 seconds. For a 100 ng/g SRM3141a solution at a flow rate of 50 µL/min, the signals on masses 39 and 41 were ~50 V and 4 V, respectively.

Evaluation of K isotopic analysis with the Neoma MS/MS was performed by measuring lunar soils and well-characterized geostandards. Each lunar soil sample was measured twice (two analyses of 75 cycles), bracketed by reference standard SRM3141a (Data S1F). Geostandards consisted of BCR-1 (basalt, USGS), JB-1 (basalt, GSJ), and G-2 (granite, USGS) as well as a

Hawaiian seawater sample that were previously analyzed for their K isotopic compositions by several laboratories using different methods (MC-ICP-MS and CC-MC-ICP-MS) (*59*, *60*, *66*, *71*).

The lunar soil data are summarized in Data S1F. The original concentrations were highly mismatched and use of a syringe injection system did not fully alleviate this mismatch, so the samples were not perfectly matched in intensity with the bracketing reference standard. For each lunar soil sample, we used the two measurements (from two different sessions) to quantify the effect of concentration mismatch. We calculated the slope of the effect of concentration mismatch (K isotopic compositions as the dependent variable and concentration mismatch as the independent variable; Data S1F) and used the intercept to represent the corrected K isotopic composition of the sample, because it corresponds to a concentration mismatch of zero. Overall, we obtained slopes ranging from −0.003 to −0.025, with an average of −0.007, corresponding to 7ppm correction for 1% concentration mismatch. This is comparable to the effect of concentration mismatch found in previous K isotopic measurements using CC-MC-ICP-MS (*62*, *65*–*67*). After correction, the K isotopic compositions are comparable to the values obtained using the Neptune Plus MC-ICP-MS (fig. S10). Because each lunar soil sample was measured only twice, and because the precision of the measurements was not as good as the measurements with the Neptune Plus MC-ICP-MS, we used the K data measured by Neptune Plus (Data S1A) when constraining the Rb-K isotopic slope. Using the K isotopic composition measured by Neoma MS/MS will not change the slope, as we calculated the K-Rb isotopic slope to be 0.170±0.041 and 0.172±0.045 using Neoma MS/MS and Neptune Plus data, respectively. For geostandards, each sample was analyzed 4 or 5 times. The values before and after correction for concentration mismatch using a slope of −0.007 constrained by lunar soil samples are shown in Data S1E. The concentration match for geostandards is more accurate than that for lunar soils and the correction is minor. The measured values agree well with literature data (Data S1G; fig. S10). The main advantage of CC-MC-ICP-MS/MS over MC-ICP-MS for K isotopic analysis is that the former uses much less K (100 ng for one measurement) compared to the latter (2000 ng for one measurement).

Elemental concentrations of K and Rb in lunar soils reflect magmatic differentiation

The effect of regolith processing is uniquely registered in the isotopic compositions of K and Rb and is not seen in the elemental concentrations (fig. S3). An enrichment of heavy isotopes of K and Rb in lunar soil regolith suggests a loss of these elements from the lunar surface. During liberation of atoms, kinetic isotope fractionation would preferentially release lighter isotopes due to their reduced masses, leaving the residue enriched in heavier isotopes. Thus, one would expect heavier isotopic compositions of lunar soils to be correlated with lower elemental concentrations. However, plotting isotopic compositions of K and Rb against their elemental concentrations shows no such correlation (fig. S3A and B; Data S1A). Instead, plotting the K and Rb elemental concentrations against U and Ba concentrations shows tight correlations (fig. S3C and D; Data S1A). Elements U and Ba are similarly incompatible and lithophile compared to K and Rb (but are more refractory than K and Rb) (*23*), thus the correlations suggest that magmatic differentiation governs variations in elemental concentrations, and the elemental loss associated with regolith processing is too small to be detected.

Lunar magmatic differentiation has led to the formation of various igneous components and terranes with contrasting alkali contents. Among them, the geochemical KREEP (K, REE, and P) component (residual melt of lunar magma ocean crystallization) is highly enriched in

incompatible elements K and Rb, while early crystallized ultramafic rocks and their derivative basalts are highly depleted in the two elements. Thus, soils that have incorporated more KREEP component would have higher K and Rb elemental contents compared to those with little KREEP contributions, explaining the large variations in K and Rb elemental concentrations (ranging between 1000–5000 µg/g for K and 5–25 µg/g for Rb; Data S1A). The dominance of magmatic differentiation over elemental loss from lunar surface in controlling the Rb and K elemental concentrations and correlation suggests that elemental concentrations cannot be used to study lunar surface weathering. The large isotopic variations of K and Rb are caused by regolith processing and provide an ideal tracer of element loss from the lunar surface by space weathering.

<u>Calculations of isotopic fractionations during meteorite impact vaporization (IV), ion sputtering (IS), photon-stimulated desorption (PSD), and the associated gravitational escape</u>

Meteorite impact vaporization (IV). Through the Hertz-Knudsen equation, the kinetic theory of gases provides a theoretical framework for understanding how K or Rb isotopes fractionate during evaporation under near vacuum conditions relevant to the lunar surface (*38*, *72–75*). Previous work examined the evaporation kinetics and isotopic fractionation of K during vaporization of silicate melts by heating (*37–39*, *76–78*). Evaporation experiments (*37*, *38*) show that to a good approximation, evaporation of K follows a Rayleigh distillation with a vapor-residue instantaneous fractionation factor of $\alpha_{\mathrm{K}} = R_{\mathrm{vapor,K}}/R_{\mathrm{residue,K}} = (39/41)^{\beta}$, with R the $^{41}\mathrm{K}/^{39}\mathrm{K}$ ratio and β close to the ideal value of 0.5. The most recent experimental study of K also studied Rb, showing that K and Rb evaporated under vacuum conditions have β values of 0.45 and 0.44, respectively (*39*), corresponding to isotopic differences between the evaporated atoms and the residue of −22.3 ‰ for K and −10.2 ‰ for Rb (Data S1C).

Gravitational escape associated with IV is often modeled as thermal escape (Eq. 1) (*30*, *32*, *43*, *47*). Micrometeorite impacts cause thermal vaporization of elements, and the temperature of the vapor was calculated based on hypervelocity particle impact experiments to be ~2500–5000 K depending on the impact velocity (*48*, *49*). At those high temperatures, a fraction of the atoms released from the lunar surface by IV can have velocities higher than the lunar escape velocity and are directly lost to space. The velocity distribution of the atoms can be parameterized using Maxwell-Boltzmann equation, which takes the form,

$$f_{\mathrm{IV}}(v) = \left(\frac{2}{\pi}\right)^{1/2} \left(\frac{m}{k_{\mathrm{B}}\,T}\right)^{3/2} v^2\, \mathrm{e}^{-\frac{mv^2}{2k_{\mathrm{B}}\,T}}, \quad (1)$$

where v is the speed of a particle in m s^{-1}, $f(v)$ is the probability distribution function of speed in s m^{-1}, m is the particle mass in kg, k_B is the Boltzmann constant 1.38×10^{-23} m^2 kg s^{-2} K^{-1}, and T is the thermodynamic (absolute) temperature in Kelvin. The speed v is a scalar quantity. An element or isotope is assumed lost to space if its speed is higher than the lunar escape velocity v_{esc} (2375 m s^{-1}). The fraction of atoms with speed above the lunar escape velocity ($F_{\mathrm{esc,IV}}$) can be derived from Eq. 1,

$$F_{\mathrm{esc,IV}} = 1 - \int_0^{v_{\mathrm{esc}}} f_{\mathrm{IV}}(v)\, dv = 1 + v_{\mathrm{esc}} \sqrt{\frac{2m}{\pi k_{\mathrm{B}}\,T}}\, \mathrm{e}^{-\frac{m\,{v_{\mathrm{esc}}}^2}{2k_{\mathrm{B}}\,T}} - Erf\left(v_{\mathrm{esc}} \sqrt{\frac{m}{2k_{\mathrm{B}}\,T}}\right). \quad (2)$$

By applying the appropriate masses of isotopes of an element, the escaped and remained fractions of each isotope can be calculated. The isotope fractionation factor (α) between escaped atoms and the bulk vapor released by IV for an element (taking K as an example) is,

$$\alpha_{\mathrm{esc-IV,K}} = \frac{R_{\mathrm{esc,IV,K}}}{R_{\mathrm{IV,K}}} = \frac{F_{\mathrm{esc,IV,41K}}}{F_{\mathrm{esc,IV,39K}}}, \ (3)$$

and the isotopic difference would be

$$\Delta_{\mathrm{esc-IV,K}} = \delta_{\mathrm{esc,IV,K}} - \delta_{\mathrm{IV,K}} = 1000\left(\alpha_{\mathrm{esc-IV,K}} - 1\right) = 1000\left(\frac{F_{\mathrm{esc,IV,41K}}}{F_{\mathrm{esc,IV,39K}}} - 1\right). \ (4)$$

The isotopic difference between the escaped atoms and the remained atoms can also be calculated,

$$\alpha_{\mathrm{esc-rem,IV,K}} = \frac{R_{\mathrm{esc,IV,K}}}{R_{\mathrm{rem,IV,K}}} = \frac{F_{\mathrm{esc,IV,41K}}}{F_{\mathrm{esc,IV,39K}}} / \frac{1-F_{\mathrm{esc,IV,41K}}}{1-F_{\mathrm{esc,IV,39K}}}, \ (5)$$

$$\Delta_{\mathrm{esc-rem,IV,K}} = 1000\left(\frac{F_{\mathrm{esc,IV,41K}}}{F_{\mathrm{esc,IV,39K}}} / \frac{1-F_{\mathrm{esc,IV,41K}}}{1-F_{\mathrm{esc,IV,39K}}} - 1\right). \ (6)$$

The escaped fractions of K and Rb during gravitational escape associated with IV as a function of temperature (1000–6000 K) are shown in fig. S4A, and the isotopic difference between the escaped atoms and the remained atoms is shown in fig. S4B. Higher temperature leads to more gravitational escape and consequently smaller isotopic difference. Although the escaped fractions are overall small, the isotope fractionations are substantial. For example, at a characteristic temperature of 4000 K (*30*, *43*), the escaped fractions of K and Rb are 8.5% and 0.2% of vaporized K and Rb atoms, respectively, but with highly fractionated isotopic compositions. The isotopic differences between escaped atoms and the bulk vapor are −139 ‰ for K and −147 ‰ for Rb, and between escaped and remaining atoms are −150 ‰ and −147 ‰ for K and Rb, respectively.

Ion sputtering (IS). The theory of ion sputtering for low-density "linear" cascades (the sputtering yield increases linearly with the energy density deposited in the surface region of the cascade) has been well established (*41*, *42*). The sputter yield ratio of two elements A and B ($Y_{\mathrm{A}}/Y_{\mathrm{B}}$) can be expressed as (*42*),

$$\frac{Y_{\mathrm{A}}}{Y_{\mathrm{B}}} = \frac{N_{\mathrm{A}}}{N_{\mathrm{B}}}\left(\frac{M_{\mathrm{B}}}{M_{\mathrm{A}}}\right)^{2\epsilon}\left(\frac{U_{\mathrm{B}}}{U_{\mathrm{A}}}\right)^{1-2\epsilon}. \ (7)$$

where N, M, and U are the number density within the sputtered material, atomic mass, and surface binding energy, respectively. ϵ is a parameter that has a value of $0 \leq \epsilon < 1$, which comes from a power-law approximation to the interaction potential between atoms. A value of $\epsilon \simeq 0.055$ corresponding to Born-Mayer interaction is the preferred value (*42*). Assuming that the two isotopes i and j of an element have the same binding energy, the isotope fractionation factor between the sputtered atoms and the original material is,

$$\alpha_{\mathrm{IS}}^{\mathrm{i/j}} = \frac{Y_{\mathrm{i}}}{Y_{\mathrm{j}}} / \frac{N_{\mathrm{i}}}{N_{\mathrm{j}}} = \left(\frac{M_{\mathrm{j}}}{M_{\mathrm{i}}}\right)^{2\epsilon}. \ (8)$$

According to Eq. 8, the K and Rb isotopic fractionations during ion sputtering of lunar soil regolith are (Data S1C),

$$\Delta_{\mathrm{IS-soil,K}}(‰) = 1000\left(\alpha_{\mathrm{IS}}^{41/39} - 1\right) = -5.49, \ (9)$$
$$\Delta_{\mathrm{IS-soil,Rb}}(‰) = 1000\left(\alpha_{\mathrm{IS}}^{87/85} - 1\right) = -2.55. \ (10)$$

Gravitational escape of sputtered K and Rb atoms can be calculated using the energy distribution of the sputtered atoms (*30*, *41*, *43*),

$$f_{\mathrm{IS}}(E_{\mathrm{e}}) = \frac{6E_{\mathrm{b}}}{3-8\sqrt{E_{\mathrm{b}}/E_{\mathrm{c}}}} \frac{E_{\mathrm{e}}}{(E_{\mathrm{e}}+E_{\mathrm{b}})^3}\left(1-\sqrt{\frac{E_{\mathrm{e}}+E_{\mathrm{b}}}{E_{\mathrm{c}}}}\right). \ (11)$$

In the above equation, E_{e} is the energy of the sputtered atoms and $f_{\mathrm{IS}}(E_{\mathrm{e}})$ is the energy distribution function, E_{b} is the surface binding energy of the sputtered atoms. The surface biding energies of K and Rb are assumed to be 2.55 eV and 2.42 eV, respectively (*79*). E_{c} is the cut-off energy and is parameterized as (*43*),

$$E_{\mathrm{c}} = E_{\mathrm{i}} \frac{4A_1A_2}{(A_1+A_2)^2}, \ (12)$$

where E_{i} is the energy of the projectile. The projectiles are mainly protons during ion sputtering of lunar surface, and are assumed to have incident energy of 1 keV (*80*). A_1 and A_2 are the atomic numbers of the projectile and target atoms, respectively. From the energy distribution one can derive the speed distribution, considering that $E_{\mathrm{e}} = \frac{1}{2}mv^2$,

$$f_{\mathrm{IS}}(v) = \frac{6E_{\mathrm{b}}Jm^2v^3\left[4-(A_1+A_2)\sqrt{\frac{4E_{\mathrm{b}}J+2mv^2}{E_{\mathrm{i}}JA_1A_2}}\right]}{(2E_{\mathrm{b}}J+mv^2)^3\left[3-4(A_1+A_2)\sqrt{\frac{E_{\mathrm{b}}}{E_{\mathrm{i}}A_1A_2}}\right]}, \ (13)$$

where m and v are the mass and speed of particles (or isotopes), respectively, and J is for converting the energy unit eV to SI unit Joule and has the value of 1.602×10^{-19} eV J^{-1}. The speed distributions of K and Rb isotopes during ion sputtering are shown in fig. S5. The escaped fraction of atoms (atoms with speed higher than the lunar escape velocity) can be integrated numerically using the above equation. We also derived the analytical solution, which takes the form,

$$F_{\mathrm{esc,IS}} = 1 - \int_0^{v_{\mathrm{esc}}} f_{\mathrm{IS}}(v)\, dv =$$
$$1 - \frac{1}{3-4(A_1+A_2)\sqrt{\frac{E_{\mathrm{b}}}{E_{\mathrm{i}}A_1A_2}}}\left(\frac{3m^2{v_{\mathrm{esc}}}^4}{(2E_{\mathrm{b}}J+m{v_{\mathrm{esc}}}^2)^2} - \frac{4E_{\mathrm{b}}(A_1+A_2)\sqrt{J}\left[-4E_{\mathrm{b}}J-3m{v_{\mathrm{esc}}}^2+(2E_{\mathrm{b}}J+m{v_{\mathrm{esc}}}^2)\sqrt{\frac{2m{v_{\mathrm{esc}}}^2}{E_{\mathrm{b}}J}+4}\right]}{\sqrt{2E_{\mathrm{i}}A_1A_2}\,(2E_{\mathrm{b}}J+m{v_{\mathrm{esc}}}^2)^{3/2}}\right). \ (14)$$

The escaped and remained fractions of each isotope can be calculated using the above equation. The isotopic fractionation factors can be calculated in the same way as those calculated for IV (Eqs. 3, 4, 5, and 6),

$$\alpha_{\mathrm{esc-IS,K}} = \frac{R_{\mathrm{esc,IS,K}}}{R_{\mathrm{IS,K}}} = \frac{F_{\mathrm{esc,IS,41K}}}{F_{\mathrm{esc,IS,39K}}}, (15)$$

$$\alpha_{\mathrm{esc-rem,IS,K}} = \frac{R_{\mathrm{esc,IS,K}}}{R_{\mathrm{rem,IS,K}}} = \frac{F_{\mathrm{esc,IS,41K}}}{F_{\mathrm{esc,IS,39K}}} / \frac{1-F_{\mathrm{esc,IS,41K}}}{1-F_{\mathrm{esc,IS,39K}}}. (16)$$

The values of the parameters for calculating K and Rb isotopic fractionations during IS and the results are summarized in Data S1C. Overall, IS causes more atoms to escape the lunar gravity compared to IV. The escaped K and Rb atoms are 88 % and 65 % of sputtered atoms, respectively, with light isotopic compositions of −9.6 ‰ for K and −12.1 ‰ for Rb relative to the bulk vapor composition, and of −75 ‰ and −33 ‰ relative to the remaining atoms.

Photon-stimulated desorption (PSD). Despite the fact that PSD has been discussed as an important process for releasing Na and K from lunar surface into the exosphere (*24*, *26*), PSD primarily release atoms that are adsorbed on the lunar surface, meaning atoms that are already freed from mineral bonds. For elements that are chemically bound within a mineral, solar photons do not have enough energy to release them efficiently (*30*). Therefore, although PSD can release surface atoms into lunar exosphere, this contribution is mainly through recycling of deposited surface atoms that were previously released by IV or IS processes. During PSD, an electronic transition happens due to photo-excitation, leading to an anti-bonding state of an atom that is then released. The process is non-thermal and is not expected to induce large isotopic fractionation.

The fraction of the PSD-released atoms that could escape lunar exosphere was calculated using the velocity distribution of the released atoms. The released Na atoms by PSD have velocity distribution functions resembling a thermal Maxwell-Boltzmann distribution at ~1200 K, but with a high-energy tail and a positive non-zero lowest velocity (*25*, *27*). It was shown that this type of velocity distribution can best be described by a two parameter (v_0 and κ) Weibull distribution (*44*),

$$f_{\mathrm{PSD}}(v) = \kappa\, \lambda^{\kappa}(v - v_0)^{\kappa-1} e^{-\lambda^{\kappa}(v-v_0)^{\kappa}};\ \lambda = \sqrt{\frac{m}{3k_{\mathrm{B}}T_{\mathrm{S}}}}\,\Gamma\left(1 + \frac{1}{\kappa}\right), (17)$$

where κ is the shape parameter ($\kappa = 1.7$), v_0 is the offset speed ($v_0 = 575$ m s^{-1}), T_{S} is the surface temperature ($T_{\mathrm{S}} = 140 - 400$ K for the Moon), k_{B} is the Boltzmann constant, and m is the mass of the desorbed atom. We used a temperature T_{S} of 400 K to calculate the maximum fraction of escaped atoms. The escaped fraction can be written as,

$$F_{\mathrm{esc,PSD}} = 1 - \int_{v_0}^{v_{\mathrm{esc}}} f_{\mathrm{PSD}}(v)\, dv = e^{-\lambda^{\kappa}(v_{esc}-v_0)^{\kappa}}. (18)$$

The values of the parameters for calculating the Rb and K isotopic fractionations during PSD-induced gravitational escape, and the calculated isotope fractionations and escaped

fractions of K and Rb are given in Data S1C. The escaped fractions of K and Rb are 8×10^{-4} and 1×10^{-6}, respectively, too small to cause any change to the elemental and isotopic compositions of lunar exosphere.

<u>Calculation of the relative contributions of sources and sinks of lunar atmosphere using the observed K-Rb isotopic slope</u>

Atoms in lunar exosphere originate from meteorite impact-induced vaporization (IV), ion sputtering (IS), and photon-stimulated desorption (PSD). Atoms in the exosphere are removed by gravitational escape, photoionization, and trapping on the lunar surface. The processes are depicted in Fig. 1. Photoionization loss occurs when exosphere atoms are ionized by solar irradiation and then either get picked up by solar wind and Earth's magnetosphere, or get re-implanted in the lunar regolith. Surface trapping happens when exosphere atoms fall back on the lunar surface and a fraction of them are permanently trapped because they are in a shadow or are covered by the ejecta of a local impact. A fraction of the atoms that fall back on the lunar surface could be recycled into the exosphere by PSD or thermal desorption because they are only weakly adsorbed on exposed surfaces or can diffuse out of the shadow trap (*56*). Those atoms can also hop several times before they are permanently removed from the exosphere (lost to space or permanently trapped on lunar surface). On the long term, the net flux of hoping atoms to the exosphere composition is null and these do not appear in the mass balance equations given below.

The contribution of each process to the lunar exosphere has been a long debate. Here we build a simple model using K and Rb isotopes to constrain the long-term relative contributions of these processes more accurately. The rate of variation in the abundance of atoms in the exosphere is determined by the difference between the input fluxes and the output fluxes (taking K as an example),

$$\frac{d\mathrm{K}_{\mathrm{atm}}}{dt} = \phi_{\mathrm{IV}} + \phi_{\mathrm{IS}} + \phi_{\mathrm{PSD}} - \phi_{\mathrm{IV,esc}} - \phi_{\mathrm{IS,esc}} - \phi_{\mathrm{PSD,esc}} - \phi_{\mathrm{i}} - \phi_{\mathrm{tr}}, \quad (19)$$

where ϕ denotes flux, atm, IV, IS, PSD, esc, i, and tr stand for atmosphere, impact-vaporized, ion-sputtered, photo-desorbed, gravitationally escaped, photoionized (either lost to space or reimplanted on the lunar surface), and surface trapped atoms (atoms from lunar atmosphere that are not photoionized but are returned to lunar soil regolith permanently). K stands for the abundance of ^{39}K and t is time. At steady state we have,

$$\phi_{\mathrm{IV}} + \phi_{\mathrm{IS}} + \phi_{\mathrm{PSD}} - \phi_{\mathrm{IV,esc}} - \phi_{\mathrm{IS,esc}} - \phi_{\mathrm{PSD,esc}} - \phi_{\mathrm{i}} - \phi_{\mathrm{tr}} = 0. \quad (20)$$

For the isotopic ratio $R = \frac{^{41}\mathrm{K}}{^{39}\mathrm{K}}$, one can write,

$$\frac{d\mathrm{K}_{\mathrm{atm}}R_{\mathrm{atm}}}{dt} = \phi_{\mathrm{IV}}R_{\mathrm{IV}} + \phi_{\mathrm{IS}}R_{\mathrm{IS}} + \phi_{\mathrm{PSD}}R_{\mathrm{PSD}} - \phi_{\mathrm{IV,esc}}R_{\mathrm{IV,esc}} - \phi_{\mathrm{IS,esc}}R_{\mathrm{IS,esc}} - \phi_{\mathrm{PSD,esc}}R_{\mathrm{PSD,esc}} - \phi_{\mathrm{i}}R_{\mathrm{i}} - \phi_{\mathrm{tr}}R_{\mathrm{tr}}. \quad (21)$$

If we note isotope fractionation factor α,

$$\alpha_{\mathrm{IV-soil}} = \frac{R_{\mathrm{IV}}}{R_{\mathrm{soil}}},$$

$$\alpha_{\text{IS-soil}} = \frac{R_{\text{IS}}}{R_{\text{soil}}},$$
$$\alpha_{\text{PSD-soil}} = \frac{R_{\text{PSD}}}{R_{\text{soil}}},$$
$$\alpha_{\text{esc-IV}} = \frac{R_{\text{IV,esc}}}{R_{\text{IV}}}, \quad (22)$$
$$\alpha_{\text{esc-PSD}} = \frac{R_{\text{PSD,esc}}}{R_{\text{PSD}}},$$
$$\alpha_{\text{esc-IS}} = \frac{R_{\text{IS,esc}}}{R_{\text{IS}}},$$
$$\alpha_{\text{i-atm}} = \frac{R_{\text{i}}}{R_{\text{atm}}},$$
$$\alpha_{\text{tr-atm}} = \frac{R_{\text{tr}}}{R_{\text{atm}}},$$

by inserting Eq. 22 into Eq. 21 and after some rearrangement, we have at steady state ($d\text{K}_{\text{atm}}R_{\text{atm}}/dt = 0$),

$$R_{\text{atm}} = \frac{R_{\text{soil}}\left(\phi_{\text{IV}}\alpha_{\text{IV-soil}}+\phi_{\text{IS}}\alpha_{\text{IS-soil}}+\phi_{\text{PSD}}\alpha_{\text{PSD-soil}}-\phi_{\text{IV,esc}}\alpha_{\text{esc-IV}}\alpha_{\text{IV-soil}}-\phi_{\text{IS,esc}}\alpha_{\text{esc-IS}}\alpha_{\text{IS-soil}}-\phi_{\text{PSD,esc}}\alpha_{\text{esc-PSD}}\alpha_{\text{PSD-soil}}\right)}{\phi_{\text{i}}\alpha_{\text{i-atm}}+\phi_{\text{tr}}\alpha_{\text{tr-atm}}}. \quad (23)$$

For the soil reservoir, we can write the following mass balance differential equation,

$$\frac{d\text{K}_{\text{soil}}}{dt} = \phi_{\text{met}} + \phi_{\text{Br}} - \phi_{\text{IV}} - \phi_{\text{IS}} - \phi_{\text{PSD}} + g\phi_{\text{i}} + \phi_{\text{tr}}, \quad (24)$$

where met is the meteoritic input to the lunar soil, Br stands for bedrocks that are added to the soil by gardening, and g denotes the fraction of photoionized atoms that are reimplanted in lunar soil (as opposed to loss to space after photoionization). The mass balance for isotopic ratios in the soil reservoir takes the form,

$$\frac{d\text{K}_{\text{soil}}R_{\text{soil}}}{dt} = \phi_{\text{met}}R_{\text{met}} + \phi_{\text{Br}}R_{\text{Br}} - \phi_{\text{IV}}R_{\text{IV}} - \phi_{\text{IS}}R_{\text{IS}} - \phi_{\text{PSD}}R_{\text{PSD}} + g\phi_{\text{i}}R_{\text{i,g}} + \phi_{\text{tr}}R_{\text{tr}}, \quad (25)$$

where R_{met} and R_{Br} are the isotopic ratios of the meteorites and bedrocks, respectively, and $R_{\text{i,g}}$ the isotopic ratio of the atoms that are photoionized and reimplanted on the lunar surface. By expanding the left side of Eq. 25 using $d\text{K}_{\text{soil}}R_{\text{soil}}/dt = R_{\text{soil}}\, d\text{K}_{\text{soil}}/dt + \text{K}_{\text{soil}}\, dR_{\text{soil}}/dt$, and combining it with Eq. 24, we have,

$$\text{K}_{\text{soil}}\frac{dlnR_{\text{soil}}}{dt} = \phi_{\text{met}}\left(\frac{R_{\text{met}}}{R_{\text{soil}}} - 1\right) + \phi_{\text{Br}}\left(\frac{R_{\text{Br}}}{R_{\text{soil}}} - 1\right) + \phi_{\text{IV}}(1 - \alpha_{\text{IV-soil}}) + \phi_{\text{IS}}(1 - \alpha_{\text{IS-soil}}) + \phi_{\text{PSD}}(1 - \alpha_{\text{PSD-soil}}) + \left(g\phi_{\text{i}}\alpha_{\text{i,g-atm}} + \phi_{\text{tr}}\alpha_{\text{tr-atm}}\right)\frac{R_{\text{atm}}}{R_{\text{soil}}} - g\phi_{\text{i}} - \phi_{\text{tr}}. \quad (26)$$

Injecting the relationship between R_{atm} and R_{soil} (Eq. 23), we get,

$$\text{K}_{\text{soil}}\frac{dlnR_{\text{soil}}}{dt} = \phi_{\text{met}}\left(\frac{R_{\text{met}}}{R_{\text{soil}}} - 1\right) + \phi_{\text{Br}}\left(\frac{R_{\text{Br}}}{R_{\text{soil}}} - 1\right) + \phi_{\text{IV}}(1 - \alpha_{\text{IV-soil}}) + \phi_{\text{IS}}(1 - \alpha_{\text{IS-soil}}) + \phi_{\text{PSD}}(1 - \alpha_{\text{PSD-soil}}) + \left(\frac{\phi_{\text{IV}}\alpha_{\text{IV-soil}}+\phi_{\text{IS}}\alpha_{\text{IS-soil}}+\phi_{\text{PSD}}\alpha_{\text{PSD-soil}}-\phi_{\text{IV,esc}}\alpha_{\text{esc-IV}}\alpha_{\text{IV-soil}}-\phi_{\text{IS,esc}}\alpha_{\text{esc-IS}}\alpha_{\text{IS-soil}}-\phi_{\text{PSD,esc}}\alpha_{\text{esc-PSD}}\alpha_{\text{PSD-soil}}}{\phi_{\text{i}}\alpha_{\text{i-atm}}+\phi_{\text{tr}}\alpha_{\text{tr-atm}}}\right)\left(g\phi_{i}\alpha_{i,g-atm} + \phi_{tr}\alpha_{tr-atm}\right) - g\phi_{i} - \phi_{\text{tr}}. \quad (27)$$

Photoionization (ϕ_i), photoionization-driven re-implantation ($g\phi_i$), and surface trapping (ϕ_{tr}) are not expected to fractionate isotopes much, so we have $\alpha_{\mathrm{i-atm}} = 1$, $\alpha_{\mathrm{i,g-atm}} = 1$, and $\alpha_{\mathrm{tr-atm}} = 1$. It follows that,

$$\mathrm{K}_{\mathrm{soil}} \frac{dlnR_{\mathrm{soil}}}{dt} = \phi_{\mathrm{met}} \left(\frac{R_{\mathrm{met}}}{R_{\mathrm{soil}}} - 1\right) + \phi_{\mathrm{Br}} \left(\frac{R_{\mathrm{Br}}}{R_{\mathrm{soil}}} - 1\right) + \phi_{\mathrm{IV}}(1 - \alpha_{\mathrm{IV-soil}}) + \phi_{\mathrm{IS}}(1 - \alpha_{\mathrm{IS-soil}}) + \phi_{\mathrm{PSD}}(1 - \alpha_{\mathrm{PSD-soil}}) + \left(\frac{\phi_{\mathrm{IV}}\alpha_{\mathrm{IV-soil}} + \phi_{\mathrm{IS}}\alpha_{\mathrm{IS-soil}} + \phi_{\mathrm{PSD}}\alpha_{\mathrm{PSD-soil}} - \phi_{\mathrm{IV,esc}}\alpha_{\mathrm{esc-IV}}\alpha_{\mathrm{IV-soil}} - \phi_{\mathrm{IS,esc}}\alpha_{\mathrm{esc-IS}}\alpha_{\mathrm{IS-soil}} - \phi_{\mathrm{PSD,esc}}\alpha_{\mathrm{esc-PSD}}\alpha_{\mathrm{PSD-soil}}}{\phi_{\mathrm{i}} + \phi_{\mathrm{tr}}} - 1\right)(g\phi_i + \phi_{tr}). \quad (28)$$

At steady state the different fluxes in and out of the atmosphere are balanced (Eq. 20), and we can use the relationship $\phi_{\mathrm{i}} + \phi_{\mathrm{tr}} = \phi_{\mathrm{IV}} + \phi_{\mathrm{PSD}} + \phi_{\mathrm{IS}} - \phi_{\mathrm{IV,esc}} - \phi_{\mathrm{PSD,esc}} - \phi_{\mathrm{IS,esc}}$ to rewrite Eq. 28 as,

$$\mathrm{K}_{\mathrm{soil}} \frac{dlnR_{\mathrm{soil}}}{dt} = \phi_{\mathrm{met}} \left(\frac{R_{\mathrm{met}}}{R_{\mathrm{soil}}} - 1\right) + \phi_{\mathrm{Br}} \left(\frac{R_{\mathrm{Br}}}{R_{\mathrm{soil}}} - 1\right) + \phi_{\mathrm{IV}}(1 - \alpha_{\mathrm{IV-soil}}) + \phi_{\mathrm{IS}}(1 - \alpha_{\mathrm{IS-soil}}) + \phi_{\mathrm{PSD}}(1 - \alpha_{\mathrm{PSD-soil}}) + \frac{g\phi_i + \phi_{\mathrm{tr}}}{\phi_{\mathrm{i}} + \phi_{\mathrm{tr}}}[\phi_{\mathrm{IV}}(\alpha_{\mathrm{IV-soil}} - 1) + \phi_{\mathrm{IS}}(\alpha_{\mathrm{IS-soil}} - 1) + \phi_{\mathrm{PSD}}(\alpha_{\mathrm{PSD-soil}} - 1) - \phi_{\mathrm{IV,esc}}(\alpha_{\mathrm{esc-IV}}\alpha_{\mathrm{IV-soil}} - 1) - \phi_{\mathrm{IS,esc}}(\alpha_{\mathrm{esc-IS}}\alpha_{\mathrm{IS-soil}} - 1) - \phi_{\mathrm{PSD,esc}}(\alpha_{\mathrm{esc-PSD}}\alpha_{\mathrm{PSD-soil}} - 1)]. \quad (29)$$

To reformulate this equation in $\delta = 1000(R/R_{\mathrm{std}} - 1)$ notation, we use $\delta \approx 1000\ln(R/R_{\mathrm{std}})$, $\Delta = 1000(\alpha - 1)$, and $\alpha_1\alpha_2 - 1 = (\Delta_1/1000 + 1)(\Delta_2/1000 + 1) - 1 \approx \Delta_1/1000 + \Delta_2/1000$, where R_{std} is the isotopic ratio of a reference standard,

$$\mathrm{K}_{\mathrm{soil}} \frac{d\delta_{\mathrm{soil}}}{dt} = \phi_{\mathrm{met}}(\delta_{\mathrm{met}} - \delta_{\mathrm{soil}}) + \phi_{\mathrm{Br}}(\delta_{\mathrm{Br}} - \delta_{\mathrm{soil}}) + \left(\frac{g\phi_i + \phi_{\mathrm{tr}}}{\phi_{\mathrm{i}} + \phi_{\mathrm{tr}}} - 1\right)(\phi_{\mathrm{IV}}\Delta_{\mathrm{IV-soil}} + \phi_{\mathrm{IS}}\Delta_{\mathrm{IS-soil}} + \phi_{\mathrm{PSD}}\Delta_{\mathrm{PSD-soil}}) - \frac{g\phi_i + \phi_{\mathrm{tr}}}{\phi_{\mathrm{i}} + \phi_{\mathrm{tr}}}[\phi_{\mathrm{IV,esc}}(\Delta_{\mathrm{esc-IV}} + \Delta_{\mathrm{IV-soil}}) + \phi_{\mathrm{IS,esc}}(\Delta_{\mathrm{esc-IS}} + \Delta_{\mathrm{IS-soil}}) + \phi_{\mathrm{PSD,esc}}(\Delta_{\mathrm{esc-PSD}} + \Delta_{\mathrm{PSD-soil}})]. \quad (30)$$

Introducing $\tau_{\mathrm{IV}} = \mathrm{K}_{\mathrm{soil}}/\phi_{\mathrm{IV}}$, the hypothetical vaporization timescale for the soil (the time that it would take to vaporize all atoms in the soil if no other process is at play), we have

$$\tau_{\mathrm{IV}} \frac{d\delta_{\mathrm{soil}}}{dt} = \frac{\phi_{\mathrm{met}}}{\phi_{\mathrm{IV}}}(\delta_{\mathrm{met}} - \delta_{\mathrm{soil}}) + \frac{\phi_{\mathrm{Br}}}{\phi_{\mathrm{IV}}}(\delta_{\mathrm{Br}} - \delta_{\mathrm{soil}}) + \left(\frac{g\phi_i + \phi_{\mathrm{tr}}}{\phi_{\mathrm{i}} + \phi_{\mathrm{tr}}} - 1\right)\left(\Delta_{\mathrm{IV-soil}} + \frac{\phi_{\mathrm{IS}}}{\phi_{\mathrm{IV}}}\Delta_{\mathrm{IS-soil}} + \frac{\phi_{\mathrm{PSD}}}{\phi_{\mathrm{IV}}}\Delta_{\mathrm{PSD-soil}}\right) - \frac{g\phi_i + \phi_{\mathrm{tr}}}{\phi_{\mathrm{i}} + \phi_{\mathrm{tr}}}\left[\frac{\phi_{\mathrm{IV,esc}}}{\phi_{\mathrm{IV}}}(\Delta_{\mathrm{esc-IV}} + \Delta_{\mathrm{IV-soil}}) + \frac{\phi_{\mathrm{IS,esc}}}{\phi_{\mathrm{IV}}}(\Delta_{\mathrm{esc-IS}} + \Delta_{\mathrm{IS-soil}}) + \frac{\phi_{\mathrm{PSD,esc}}}{\phi_{\mathrm{IV}}}(\Delta_{\mathrm{esc-PSD}} + \Delta_{\mathrm{PSD-soil}})\right]. \quad (31)$$

While regolith processing has a significant effect on isotopes, the effect on abundance is small (as discussed above that the elemental concentrations of lunar soils are not much affected by space weathering) and we can reasonably assume that $\tau_{\mathrm{IV}} = \mathrm{K}_{\mathrm{soil}}/\phi_{\mathrm{IV}}$ is constant. Eq. 31 is thus an ordinary differential equation of the form

$$d\delta_{\mathrm{soil}}/dt + a\delta_{\mathrm{soil}} = b,$$

with

$a = \frac{\phi_{\mathrm{met}}+\phi_{\mathrm{Br}}}{\tau_{\mathrm{IV}}\,\phi_{\mathrm{IV}}}$,

and

$b = \frac{1}{\tau_{\mathrm{IV}}}\left(\frac{\phi_{\mathrm{met}}}{\phi_{\mathrm{IV}}}\delta_{\mathrm{met}} + \frac{\phi_{\mathrm{Br}}}{\phi_{\mathrm{IV}}}\delta_{\mathrm{Br}}\right) - \frac{1}{\tau_{\mathrm{IV}}}\left(1 - \frac{g\phi_i+\phi_{\mathrm{tr}}}{\phi_{\mathrm{i}}+\phi_{\mathrm{tr}}}\right)\left(\Delta_{\mathrm{IV-soil}} + \frac{\phi_{\mathrm{IS}}}{\phi_{\mathrm{IV}}}\Delta_{\mathrm{IS-soil}} + \frac{\phi_{\mathrm{PSD}}}{\phi_{\mathrm{IV}}}\Delta_{\mathrm{PSD-soil}}\right) - \frac{1}{\tau_{\mathrm{IV}}}\frac{g\phi_i+\phi_{\mathrm{tr}}}{\phi_{\mathrm{i}}+\phi_{\mathrm{tr}}}\left[\frac{\phi_{\mathrm{IV,esc}}}{\phi_{\mathrm{IV}}}(\Delta_{\mathrm{esc-IV}} + \Delta_{\mathrm{IV-soil}}) + \frac{\phi_{\mathrm{IS,esc}}}{\phi_{\mathrm{IV}}}(\Delta_{\mathrm{esc-IS}} + \Delta_{\mathrm{IS-soil}}) + \frac{\phi_{\mathrm{PSD,esc}}}{\phi_{\mathrm{IV}}}(\Delta_{\mathrm{esc-PSD}} + \Delta_{\mathrm{PSD-soil}})\right]$. (32)

The solution to this differential equation is,

$\delta_{\mathrm{soil}} = \left(\delta_{\mathrm{soil,0}} - \frac{b}{a}\right)e^{-at} + \frac{b}{a} = \delta_{\mathrm{soil,0}}\,e^{-at} + \frac{b}{a}(1 - e^{-at})$, (33)

where $\delta_{\mathrm{soil,\,0}}$ is the δ value of lunar soil at time zero (the original lunar soil/bedrock composition). Inserting Eq. 32 into Eq. 33, we get,

$\delta_{\mathrm{soil}} = \delta_{\mathrm{soil,0}}\,e^{-\frac{t}{\tau_{\mathrm{IV}}}\left(\frac{\phi_{\mathrm{met}}+\phi_{\mathrm{Br}}}{\phi_{\mathrm{IV}}}\right)} + \left(\frac{\phi_{\mathrm{met}}\delta_{\mathrm{met}}+\phi_{\mathrm{Br}}\delta_{\mathrm{Br}}}{\phi_{\mathrm{met}}+\phi_{\mathrm{Br}}}\right)\left[1 - e^{-\frac{t}{\tau_{\mathrm{IV}}}\left(\frac{\phi_{\mathrm{met}}+\phi_{\mathrm{Br}}}{\phi_{\mathrm{IV}}}\right)}\right] - \frac{\phi_{\mathrm{IV}}}{\phi_{\mathrm{met}}+\phi_{\mathrm{Br}}}\left[1 - e^{-\frac{t}{\tau_{\mathrm{IV}}}\left(\frac{\phi_{\mathrm{met}}+\phi_{\mathrm{Br}}}{\phi_{\mathrm{IV}}}\right)}\right]\left\{\left(1 - \frac{g\phi_{\mathrm{i}}+\phi_{\mathrm{tr}}}{\phi_{\mathrm{i}}+\phi_{\mathrm{tr}}}\right)\left(\Delta_{\mathrm{IV-soil}} + \frac{\phi_{\mathrm{IS}}}{\phi_{\mathrm{IV}}}\Delta_{\mathrm{IS-soil}} + \frac{\phi_{\mathrm{PSD}}}{\phi_{\mathrm{IV}}}\Delta_{\mathrm{PSD-soil}}\right) + \frac{g\phi_{\mathrm{i}}+\phi_{\mathrm{tr}}}{\phi_{\mathrm{i}}+\phi_{\mathrm{tr}}}\left[\frac{\phi_{\mathrm{IV,esc}}}{\phi_{\mathrm{IV}}}(\Delta_{\mathrm{esc-IV}} + \Delta_{\mathrm{IV-soil}}) + \frac{\phi_{\mathrm{IS,esc}}}{\phi_{\mathrm{IV}}}(\Delta_{\mathrm{esc-IS}} + \Delta_{\mathrm{IS-soil}}) + \frac{\phi_{\mathrm{PSD,esc}}}{\phi_{\mathrm{IV}}}(\Delta_{\mathrm{esc-PSD}} + \Delta_{\mathrm{PSD-soil}})\right]\right\}$. (34)

Equation 34 gives the evolution of the isotopic composition of lunar soils as a function of time, and some assumptions can be safely made to simplify the equation. If we take the isotopic composition of the bulk silicate Moon as that of the reference standard, then $\delta_{\mathrm{soil,\,0}} = \delta_{\mathrm{Br}} = 0$. Coincidentally, the reference standards used for Rb and K isotopic measurements, SRM 984 and SRM 3141a, respectively, happen to have the same or very similar Rb and K isotopic compositions to the bulk Moon. The measured bulk silicate Moon has Rb and K isotopic compositions relative to the two standards of near zero (+0.03±0.03‰ for Rb and −0.01±0.02‰ for K (*14, 16*)). The isotopic compositions of meteorites that are added to the lunar surface (δ_{met}) could have non-zero values but they are still very close to zero compared to lunar soils. The measured Rb and K isotopic compositions of chondrites (the possible meteorites that were delivered to lunar soils) show limited variations (*16, 57, 64*) that are about an order of magnitude smaller than that observed in lunar soils (−0.1 – +2.2 ‰ for Rb and −0.2 – +12 ‰ for K; this study). Thus, one can assume that $\delta_{\mathrm{met}} \simeq \delta_{\mathrm{soil,0}} = \delta_{\mathrm{Br}} = 0$. Eqs. 33 and 34 then become,

$\delta_{\mathrm{soil}} = \frac{b}{a}(1 - e^{-at})$, (35)

$$\delta_{\text{soil}} = -\frac{\phi_{\text{IV}}}{\phi_{\text{met}}+\phi_{\text{Br}}}\left[1 - e^{-\frac{t}{\tau_{\text{IV}}}\left(\frac{\phi_{\text{met}}+\phi_{\text{Br}}}{\phi_{\text{IV}}}\right)}\right]\left\{\left(1-\frac{g\phi_{\text{i}}+\phi_{\text{tr}}}{\phi_{\text{i}}+\phi_{\text{tr}}}\right)\left(\Delta_{\text{IV}-\text{soil}} + \frac{\phi_{\text{IS}}}{\phi_{\text{IV}}}\Delta_{\text{IS}-\text{soil}} + \frac{\phi_{\text{PSD}}}{\phi_{\text{IV}}}\Delta_{\text{PSD}-\text{soil}}\right) + \frac{g\phi_{\text{i}}+\phi_{\text{tr}}}{\phi_{\text{i}}+\phi_{\text{tr}}}\left[\frac{\phi_{\text{IV,esc}}}{\phi_{\text{IV}}}(\Delta_{\text{esc}-\text{IV}} + \Delta_{\text{IV}-\text{soil}}) + \frac{\phi_{\text{IS,esc}}}{\phi_{\text{IV}}}(\Delta_{\text{esc}-\text{IS}} + \Delta_{\text{IS}-\text{soil}}) + \frac{\phi_{\text{PSD,esc}}}{\phi_{\text{IV}}}(\Delta_{\text{esc}-\text{PSD}} + \Delta_{\text{PSD}-\text{soil}})\right]\right\}. \quad (36)$$

Our measurements of lunar soils show that the isotopic compositions of Rb and K are tightly correlated along a line with a slope $\theta_{\text{Rb/K}} = \delta\text{Rb}_{\text{soil}}/\delta\text{K}_{\text{soil}} = 0.172 \pm 0.045$, and the slope does not depend on time t (Fig. 2). This could be explained by Eq. 35 if the a value is the same for K and Rb (the b value does not matter as it does not depend on time, while a matters because it is multiplied by t), as explained below. According to Eq. 35, the K-Rb isotopic slope $\theta_{\text{Rb/K}}$ can be expressed as,

$$\theta_{\text{Rb/K}} = \frac{\delta_{\text{soil,Rb}}}{\delta_{\text{soil,K}}} = \frac{b_{\text{Rb}}/b_{\text{K}}}{a_{\text{Rb}}/a_{\text{K}}}\frac{1-e^{-a_{\text{Rb}}t}}{1-e^{-a_{\text{K}}t}}, \quad (37)$$

with $a = \frac{\phi_{\text{met}}+\phi_{\text{Br}}}{\tau_{\text{IV}}\phi_{\text{IV}}}$ (Eq. 32). By definition $\tau_{\text{K,IV}} = \text{K}_{\text{soil}}/\phi_{\text{K,IV}}$, we have $\frac{\tau_{\text{IV,Rb}}\phi_{\text{IV,Rb}}}{\tau_{\text{IV,K}}\phi_{\text{IV,K}}} = \frac{\text{Rb}_{\text{soil}}}{\text{K}_{\text{soil}}}$. In meteorites, the Rb/K ratio does not vary much despite that the depletions of the two elements vary several orders of magnitude (*23*, *16*, *81*), possibly because that the two elements behave congruently during thermal vaporization (*39*). The Rb/K ratio in meteorites is similar to the ratio in lunar bedrocks and soil samples, thus $\frac{\phi_{\text{met,Rb}}}{\phi_{\text{met,K}}} \simeq \frac{\phi_{\text{Br,Rb}}}{\phi_{\text{Br,K}}} \simeq \frac{\text{Rb}_{\text{soil}}}{\text{K}_{\text{soil}}}$. Therefore, we have $a_{\text{Rb}} \simeq a_{\text{K}}$. Eq. 37 simplifies as,

$$\theta_{\text{Rb/K}} = b_{\text{Rb}}/b_{\text{K}}. \quad (38)$$

Eq. 38 shows that the Rb-K isotopic slope does not depend on time, because there is no time component involved in the equation. The main reason to this is that the Rb/K ratios in various meteorites and lunar rocks are very similar, which is possibly because that the two elements behave similarly during evaporation associated with planetary formation and accretion (*16*). This explains why the lunar soil samples from various locations with various degrees of maturity fall on the same Rb-K isotopic correlation. The complete form of the isotopic slope based on Eq. 38 is,

$$\theta_{\text{Rb/K}} = \frac{\left(1-\frac{g_{\text{Rb}}\phi_{\text{i,Rb}}+\phi_{\text{tr,Rb}}}{\phi_{\text{i,Rb}}+\phi_{\text{tr,Rb}}}\right)\left(\Delta_{\text{IV}-\text{soil,Rb}}+\frac{\phi_{\text{IS,Rb}}}{\phi_{\text{IV,Rb}}}\Delta_{\text{IS}-\text{soil,Rb}}+\frac{\phi_{\text{PSD,Rb}}}{\phi_{\text{IV,Rb}}}\Delta_{\text{PSD}-\text{soil,Rb}}\right)+\frac{g_{\text{Rb}}\phi_{\text{i,Rb}}+\phi_{\text{tr,Rb}}}{\phi_{\text{i,Rb}}+\phi_{\text{tr,Rb}}}\left[\frac{\phi_{\text{IV,esc,Rb}}}{\phi_{\text{IV,Rb}}}(\Delta_{\text{esc}-\text{IV,Rb}}+\Delta_{\text{IV}-\text{soil,Rb}})+\frac{\phi_{\text{IS,esc,Rb}}}{\phi_{\text{IV,Rb}}}(\Delta_{\text{esc}-\text{IS,Rb}}+\Delta_{\text{IS}-\text{soil,Rb}})+\frac{\phi_{\text{PSD,esc,Rb}}}{\phi_{\text{IV,Rb}}}(\Delta_{\text{esc}-\text{PSD,Rb}}+\Delta_{\text{PSD}-\text{soil,Rb}})\right]}{\left(1-\frac{g_{\text{K}}\phi_{\text{i,K}}+\phi_{\text{tr,K}}}{\phi_{\text{i,K}}+\phi_{\text{tr,K}}}\right)\left(\Delta_{\text{IV}-\text{soil,K}}+\frac{\phi_{\text{IS,K}}}{\phi_{\text{IV,K}}}\Delta_{\text{IS}-\text{soil,K}}+\frac{\phi_{\text{PSD,K}}}{\phi_{\text{IV,K}}}\Delta_{\text{PSD}-\text{soil,K}}\right)+\frac{g_{\text{K}}\phi_{\text{i,K}}+\phi_{\text{tr,K}}}{\phi_{\text{i,K}}+\phi_{\text{tr,K}}}\left[\frac{\phi_{\text{IV,esc,K}}}{\phi_{\text{IV,K}}}(\Delta_{\text{esc}-\text{IV,K}}+\Delta_{\text{IV}-\text{soil,K}})+\frac{\phi_{\text{IS,esc,K}}}{\phi_{\text{IV,K}}}(\Delta_{\text{esc}-\text{IS,K}}+\Delta_{\text{IS}-\text{soil,K}})+\frac{\phi_{\text{PSD,esc,K}}}{\phi_{\text{IV,K}}}(\Delta_{\text{esc}-\text{PSD,K}}+\Delta_{\text{PSD}-\text{soil,K}})\right]}. \quad (39)$$

This expression can be further simplified by recognizing that PSD does not cause gravitational escape due to its low energy (see calculation above and Data S1C), thus $\phi_{\text{PSD,esc,Rb}} = \phi_{\text{PSD,esc,K}} = 0$. Because it is a non-thermal, single electron transition process, release of atoms by PSD should not discriminate largely between isotopes, thus $\Delta_{\text{PSD}-\text{soil,Rb}} = \Delta_{\text{PSD}-\text{soil,K}} = 0$. The above equation then becomes,

$$\theta_{\mathrm{Rb/K}} = \frac{\left(1-\frac{g_{\mathrm{Rb}}\phi_{\mathrm{i,Rb}}+\phi_{\mathrm{tr,Rb}}}{\phi_{\mathrm{i,Rb}}+\phi_{\mathrm{tr,Rb}}}\right)\left(\Delta_{\mathrm{IV-soil,Rb}}+\frac{\phi_{\mathrm{IS,Rb}}}{\phi_{\mathrm{IV,Rb}}}\Delta_{\mathrm{IS-soil,Rb}}\right)+\frac{g_{\mathrm{Rb}}\phi_{\mathrm{i,Rb}}+\phi_{\mathrm{tr,Rb}}}{\phi_{\mathrm{i,Rb}}+\phi_{\mathrm{tr,Rb}}}\left[\frac{\phi_{\mathrm{IV,esc,Rb}}}{\phi_{\mathrm{IV,Rb}}}(\Delta_{\mathrm{esc-IV,Rb}}+\Delta_{\mathrm{IV-soil,Rb}})+\frac{\phi_{\mathrm{IS,esc,Rb}}}{\phi_{\mathrm{IV,Rb}}}(\Delta_{\mathrm{esc-IS,Rb}}+\Delta_{\mathrm{IS-soil,Rb}})\right]}{\left(1-\frac{g_{\mathrm{K}}\phi_{\mathrm{i,K}}+\phi_{\mathrm{tr,K}}}{\phi_{\mathrm{i,K}}+\phi_{\mathrm{tr,K}}}\right)\left(\Delta_{\mathrm{IV-soil,K}}+\frac{\phi_{\mathrm{IS,K}}}{\phi_{\mathrm{IV,K}}}\Delta_{\mathrm{IS-soil,K}}\right)+\frac{g_{\mathrm{K}}\phi_{\mathrm{i,K}}+\phi_{\mathrm{tr,K}}}{\phi_{\mathrm{i,K}}+\phi_{\mathrm{tr,K}}}\left[\frac{\phi_{\mathrm{IV,esc,K}}}{\phi_{\mathrm{IV,K}}}(\Delta_{\mathrm{esc-IV,K}}+\Delta_{\mathrm{IV-soil,K}})+\frac{\phi_{\mathrm{IS,esc,K}}}{\phi_{\mathrm{IV,K}}}(\Delta_{\mathrm{esc-IS,K}}+\Delta_{\mathrm{IS-soil,K}})\right]}$$
. (40)

Because photoionization is a one photon process, and the first ionization potentials of K and Rb (419 and 402 kJ/mol, respectively) are similar, their photoionization efficiencies should be similar ($\frac{\phi_{\mathrm{i,Rb}}}{\phi_{\mathrm{i,K}}} \simeq \frac{\mathrm{Rb_{atm}}}{\mathrm{K_{atm}}}$). Once an atom is photoionized, the chances for it to be picked up by solar wind and to be returned to the lunar surface are about equal (i.e., the g value should be close to 0.5 for both K and Rb (*45*)). For atoms in the atmosphere that are not photoionized but fall back to lunar surface and get trapped (ϕ_{tr}), K and Rb should not be discriminated as gravitational fallback does not depend on mass, and the efficiencies of trapping of them in shadows are also likely to be very close as it is primarily a geometrical question, unless surface diffusion plays a major role, in which case $\phi_{\mathrm{tr}} = 0$ for both K and Rb. Therefore, we can assume that $\phi_{\mathrm{tr,Rb}}$ and $\phi_{\mathrm{tr,K}}$ are proportional to the Rb and K amount in the lunar atmosphere $\frac{\phi_{\mathrm{tr,Rb}}}{\phi_{\mathrm{tr,K}}} \simeq \frac{\mathrm{Rb_{atm}}}{\mathrm{K_{atm}}}$. The above equation then becomes,

$$\theta_{\mathrm{Rb/K}} = \frac{\frac{(1-g)\phi_{\mathrm{i}}}{\phi_{\mathrm{i}}+\phi_{\mathrm{tr}}}\left(\Delta_{\mathrm{IV-soil,Rb}}+\frac{0.69\phi_{\mathrm{IS,K}}}{\phi_{\mathrm{IV,K}}}\Delta_{\mathrm{IS-soil,Rb}}\right)+\frac{g\phi_{\mathrm{i}}+\phi_{\mathrm{tr}}}{\phi_{\mathrm{i}}+\phi_{\mathrm{tr}}}\left[\frac{\phi_{\mathrm{IV,esc,Rb}}}{\phi_{\mathrm{IV,Rb}}}(\Delta_{\mathrm{esc-IV,Rb}}+\Delta_{\mathrm{IV-soil,Rb}})+\frac{\phi_{\mathrm{IS,esc,Rb}}}{\phi_{\mathrm{IS,Rb}}}\frac{0.69\phi_{\mathrm{IS,K}}}{\phi_{\mathrm{IV,K}}}(\Delta_{\mathrm{esc-IS,Rb}}+\Delta_{\mathrm{IS-soil,Rb}})\right]}{\frac{(1-g)\phi_{\mathrm{i}}}{\phi_{\mathrm{i}}+\phi_{\mathrm{tr}}}\left(\Delta_{\mathrm{IV-soil,K}}+\frac{\phi_{\mathrm{IS,K}}}{\phi_{\mathrm{IV,K}}}\Delta_{\mathrm{IS-soil,K}}\right)+\frac{g\phi_{\mathrm{i}}+\phi_{\mathrm{tr}}}{\phi_{\mathrm{i}}+\phi_{\mathrm{tr}}}\left[\frac{\phi_{\mathrm{IV,esc,K}}}{\phi_{\mathrm{IV,K}}}(\Delta_{\mathrm{esc-IV,K}}+\Delta_{\mathrm{IV-soil,K}})+\frac{\phi_{\mathrm{IS,esc,K}}}{\phi_{\mathrm{IS,K}}}\frac{\phi_{\mathrm{IS,K}}}{\phi_{\mathrm{IV,K}}}(\Delta_{\mathrm{esc-IS,K}}+\Delta_{\mathrm{IS-soil,K}})\right]},$$
(41)

with $\frac{\phi_{\mathrm{IS,Rb}}}{\phi_{\mathrm{IV,Rb}}} = 0.69\,\frac{\phi_{\mathrm{IS,K}}}{\phi_{\mathrm{IV,K}}}$ (Data S1C). The coefficient 0.69 is because ion sputtering can fractionate K from Rb, and secondary ionization mass spectrometry indicates that the sputtering yield of Rb is about 0.69 times of that of K, in contrast to impact-induced vaporization during which the two elements are evaporated in similar efficacy (*39*, *40*).

In the above equation, most terms are well constrained (Data S1C) and there are two uncertain parameters: 1) $\phi_{\mathrm{IS,K}}/\phi_{\mathrm{IV,K}}$, the relative efficacies of ion-sputtering and impact volatilization as atmospheric sources, and 2) $\phi_{\mathrm{i}}/\phi_{\mathrm{tr}}$, the relative efficacies of photoionization loss to space and surface trapping as atmospheric sinks. To investigate their possible values, we investigate values of $A = \frac{\phi_{\mathrm{IS,K}}}{\phi_{\mathrm{IS,K}}+\phi_{\mathrm{IV,K}}}$ and $B = \frac{\phi_{\mathrm{i}}}{\phi_{\mathrm{i}}+\phi_{\mathrm{tr}}}$, given that the two terms have bounds of 0 and 1. Parameter A represents the fraction of the atom flux contributed by the IS, out of the combined flux from both IS and IV as atmospheric sources. A value smaller than 0.5 would indicate that IV is the dominant source. Parameter B represents the faction of atom flux for photoionization, out of the total flux from both photoionization and surface trapping as atmospheric sinks. A value smaller than 0.5 would indicate that surface trapping is the dominant sink.

Rewriting Eq. 41 in terms of A and B, we have

$$\theta_{\mathrm{Rb/K}} = \frac{(1-g)B\left(\Delta_{\mathrm{IV-soil,Rb}}+0.69\frac{A}{1-A}\Delta_{\mathrm{IS-soil,Rb}}\right)+[1-(1-g)B]\left[\frac{\phi_{\mathrm{IV,esc,Rb}}}{\phi_{\mathrm{IV,Rb}}}(\Delta_{\mathrm{esc-IV,Rb}}+\Delta_{\mathrm{IV-soil,Rb}})+0.69\frac{\phi_{\mathrm{IS,esc,Rb}}}{\phi_{\mathrm{IS,Rb}}}\frac{A}{1-A}(\Delta_{\mathrm{esc-IS,Rb}}+\Delta_{\mathrm{IS-soil,Rb}})\right]}{(1-g)B\left(\Delta_{\mathrm{IV-soil,K}}+\frac{A}{1-A}\Delta_{\mathrm{IS-soil,K}}\right)+[1-(1-g)B]\left[\frac{\phi_{\mathrm{IV,esc,K}}}{\phi_{\mathrm{IV,K}}}(\Delta_{\mathrm{esc-IV,K}}+\Delta_{\mathrm{IV-soil,K}})+\frac{\phi_{\mathrm{IS,esc,K}}}{\phi_{\mathrm{IS,K}}}\frac{A}{1-A}(\Delta_{\mathrm{esc-IS,K}}+\Delta_{\mathrm{IS-soil,K}})\right]}.$$

(42)

Assuming a g value of 0.5 (*45*), Fig. 4A plots $\theta_{\mathrm{Rb/K}}$ as a function of $A = \frac{\phi_{\mathrm{IS,K}}}{\phi_{\mathrm{IS,K}}+\phi_{\mathrm{IV,K}}}$ and $B = \frac{\phi_{\mathrm{i}}}{\phi_{\mathrm{i}}+\phi_{\mathrm{tr}}}$ values. The observed slope of 0.172 requires that the A and B values to be between 0–0.319 and 0–0.478, respectively, and the two values are highly correlated.

We used a Markov Chain Monte Carlo (MCMC) method to determine the uncertainties of the two parameters. For MCMC sampling, we used uniform distributions as priors. We ran 1,500,000 iterations within the parameter space of 0–0.5 for A and B, and discarded the first 10% iterations (150,000 runs) as burn-in. The posterior joint probability distribution of (A, B) is shown in Fig. 4B. The high probability area (bright yellow region) is consistent with the slope curve in Fig. 4A, with a nominal mean ± standard deviation of 0.166 ± 0.090 for A and 0.257 ± 0.133 for B. However, the high probability area is rather uniform, indicating similar possibilities throughout the region.

K-Rb isotopic slopes for the endmember source-sink scenarios

From Eq. 41 one can derive the analytical equations for the slopes of the four endmember scenarios considered in the main text: IV as the only atmospheric source and photoionization as the only sink, IV as the only source and surface trapping as the only sink, IS as the only source and photoionization as the only sink, and IS as the only source and surface trapping as the only sink. Two equations can be written, one for IV as the only source without any contribution from IS and the other for IS as the only source and without any contribution from IV. If IV is the only atmospheric source, the equation for the K-Rb isotopic slope ($\theta_{\mathrm{Rb/K,IV}}$) is,

$$\theta_{\mathrm{Rb/K,IV}} = \frac{\frac{(1-g)\phi_{\mathrm{i}}}{\phi_{\mathrm{i}}+\phi_{\mathrm{tr}}}\Delta_{\mathrm{IV-soil,Rb}}+\frac{g\phi_{\mathrm{i}}+\phi_{\mathrm{tr}}}{\phi_{\mathrm{i}}+\phi_{\mathrm{tr}}}\left[\frac{\phi_{\mathrm{IV,esc,Rb}}}{\phi_{\mathrm{IV,Rb}}}(\Delta_{\mathrm{esc-IV,Rb}}+\Delta_{\mathrm{IV-soil,Rb}})\right]}{\frac{(1-g)\phi_{\mathrm{i}}}{\phi_{\mathrm{i}}+\phi_{\mathrm{tr}}}\Delta_{\mathrm{IV-soil,K}}+\frac{g\phi_{\mathrm{i}}+\phi_{\mathrm{tr}}}{\phi_{\mathrm{i}}+\phi_{\mathrm{tr}}}\left[\frac{\phi_{\mathrm{IV,esc,K}}}{\phi_{\mathrm{IV,K}}}(\Delta_{\mathrm{esc-IV,K}}+\Delta_{\mathrm{IV-soil,K}})\right]}. \quad (43)$$

$\frac{\phi_{\mathrm{i}}}{\phi_{\mathrm{i}}+\phi_{\mathrm{tr}}} = 1$ corresponds to photoionization as the only sink of the atmospheric atoms (no surface trapping), while $\frac{\phi_{\mathrm{i}}}{\phi_{\mathrm{i}}+\phi_{\mathrm{tr}}} = 0$ corresponds to surface trapping as the only sink (no photoionization). It can be seen from the above equation that when $\frac{\phi_{\mathrm{i}}}{\phi_{\mathrm{i}}+\phi_{\mathrm{tr}}} = 0$ (surface trapping as the sink), the slope is mainly controlled by isotope fractionation associated with gravitational escape ($\Delta_{\mathrm{esc-IV}}$) (and isotope fractionation by impact vaporization ($\Delta_{\mathrm{IV-soil}}$) is much smaller). While for photoionization as the sink $\frac{\phi_{\mathrm{i}}}{\phi_{\mathrm{i}}+\phi_{\mathrm{tr}}} = 1$, and assuming a g value of 0.5, more contribution of isotopic fractionation by impact vaporization ($\Delta_{\mathrm{IV-soil}}$) to the slope is expected.

Similarly for IS, the equation describing the K-Rb isotopic slope ($\theta_{\mathrm{Rb/K,IS}}$) can be written as,

$$\theta_{\mathrm{Rb/K,IS}} = \frac{\frac{0.69(1-g)\phi_\mathrm{i}}{\phi_\mathrm{i}+\phi_\mathrm{tr}}\Delta_{\mathrm{IS-soil,Rb}} + \frac{0.69(g\phi_\mathrm{i}+\phi_\mathrm{tr})}{\phi_\mathrm{i}+\phi_\mathrm{tr}}\left[\frac{\phi_{\mathrm{IS,esc,Rb}}}{\phi_{\mathrm{IS,Rb}}}\left(\Delta_{\mathrm{esc-IS,Rb}}+\Delta_{\mathrm{IS-soil,Rb}}\right)\right]}{\frac{(1-g)\phi_\mathrm{i}}{\phi_\mathrm{i}+\phi_\mathrm{tr}}\Delta_{\mathrm{IS-soil,K}} + \frac{g\phi_\mathrm{i}+\phi_\mathrm{tr}}{\phi_\mathrm{i}+\phi_\mathrm{tr}}\left[\frac{\phi_{\mathrm{IS,esc,K}}}{\phi_{\mathrm{IS,K}}}\left(\Delta_{\mathrm{esc-IS,K}}+\Delta_{\mathrm{IS-soil,K}}\right)\right]}. \quad (44)$$

One can set $\frac{\phi_\mathrm{i}}{\phi_\mathrm{i}+\phi_\mathrm{tr}} = 1$ for photoionization as the sink, and $\frac{\phi_\mathrm{i}}{\phi_\mathrm{i}+\phi_\mathrm{tr}} = 0$ for surface trapping as the sink. The factor 0.69 takes into account that the sputtering yield of Rb is about 30% lower than that of K (*40*).

<u>The probability of atom loss does not change over atom jumps</u>

When atoms are released from the lunar surface by IS or IV, a fraction of them are directly lost to space due to gravitational escape. The remaining atoms can jump multiple times before they are eventually photoionized or trapped on the lunar surface. For atoms that are photoionized, they can either be taken up by solar wind and lost to space or be reimplanted on the lunar surface.

During each jump, the probabilities that the atoms are trapped permanently in shadows (surface trapping) or photoionized are p_{tr} and p_{pi}, respectively. A fraction g of the photoionized atoms are reimplanted on the lunar surface while a fraction $1-g$ are lost to space. If we start with 1 atom, there will be $\left(1-p_{tr}-p_{pi}\right)^{i-1}$ remaining at step i. The fates of atoms at step i are as follows: trapped = $p_{tr}\left(1-p_{tr}-p_{pi}\right)^{i-1}$, photoionized and implanted = $gp_{pi}\left(1-p_{tr}-p_{pi}\right)^{i-1}$, photoionized and lost to space = $(1-g)p_{pi}\left(1-p_{tr}-p_{pi}\right)^{i-1}$. The grand totals for the different fractions over all jumps are,

$$f_{tr} = p_{tr}\sum_{i=1}^{\infty}\left(1-p_{tr}-p_{pi}\right)^{i-1} = \frac{p_{tr}}{p_{tr}+p_{pi}}, \quad (45)$$

$$f_{pi,implanted} = gp_{pi}\sum_{i=1}^{\infty}\left(1-p_{tr}-p_{pi}\right)^{i-1} = \frac{gp_{pi}}{p_{tr}+p_{pi}}, \quad (46)$$

$$f_{pi,lost} = (1-g)p_{pi}\sum_{i=1}^{\infty}\left(1-p_{tr}-p_{pi}\right)^{i-1} = \frac{(1-g)p_{pi}}{p_{tr}+p_{pi}}. \quad (47)$$

The above calculations show that even if loss of exospheric atoms happens over multiple jumps, the fractions that are trapped, photoionized and lost to space, and photoionized and implanted are given by the relative probabilities of these processes in any step.

<u>The effect of degree of vaporization during IV on the calculated atmospheric sources and sinks</u>

An uncertainty associated with the model calculations is the degree of evaporation of elements from the lunar surface to the lunar atmosphere during IV. Kinetic effects impart strong isotopic fractionation to the original vapor (lighter isotopes evaporate faster than heavier ones), but as evaporation proceeds and more atoms are transferred to the vapor, its isotopic composition evolves towards the initial regolith composition. Therefore, as evaporation proceeds, the isotopic fractionation between the vapor and the bulk soil would become smaller. We assumed minimal vaporization in our calculations for Fig. 4, which corresponds to the largest isotopic fractionation conceivable (−22 ‰ for K and −10 ‰ for Rb). Here we calculate the relative contributions of the

atmospheric sources and sinks ($\frac{\phi_{\mathrm{IS,K}}}{\phi_{\mathrm{IS,K}}+\phi_{\mathrm{IV,K}}}$ and $\frac{\phi_{\mathrm{i}}}{\phi_{\mathrm{i}}+\phi_{\mathrm{tr}}}$ values) assuming various degrees of evaporation (f_{evp}), which corresponds to various isotopic differences between the vapor and the bulk soil regolith. In a simplified Rayleigh distillation scenario, an f_{evp} value of close to zero means minimal evaporation and maximum isotopic fractionation between vapor and bulk soil regolith, and an f_{evp} value of 1 corresponds to fully evaporation of impacted material and thus no isotopic fractionation.

Another way to consider the isotopic difference between the vapor and the bulk soil is to consider mixing between isotopically fractionated and unfractionated vapors produced during impact vaporization. A fraction of impacted material may fully vaporize, not producing much isotopic fractionation. Other impacted material could experience partial evaporation, resulting in isotopic fractionation. The isotopic fractionation between the vapor and the bulk soil is therefore uncertain and could be influenced by mixing between vapors from variably affected evaporated materials. In all cases, the isotopic fractionation between the vapor and the bulk soil will range between zero and the maximum isotopic fractionation of −22 ‰ for K and −10 ‰ for Rb.

Below, we calculate how the isotopic fractionation between vapor and bulk soil regolith changes the calculated $\frac{\phi_{\mathrm{IS,K}}}{\phi_{\mathrm{IS,K}}+\phi_{\mathrm{IV,K}}}$ and $\frac{\phi_{\mathrm{i}}}{\phi_{\mathrm{i}}+\phi_{\mathrm{tr}}}$ values. We first calculate the isotopic fractionation as a function of the degree of evaporation f_{evp} using a simple Rayleigh distillation model. We then incorporate the calculated isotopic fractionations into Eq. 42 to calculate the values of $\frac{\phi_{\mathrm{IS,K}}}{\phi_{\mathrm{IS,K}}+\phi_{\mathrm{IV,K}}}$ and $\frac{\phi_{\mathrm{i}}}{\phi_{\mathrm{i}}+\phi_{\mathrm{tr}}}$.

The calculated $\frac{\phi_{\mathrm{IS,K}}}{\phi_{\mathrm{IS,K}}+\phi_{\mathrm{IV,K}}}$ and $\frac{\phi_{\mathrm{i}}}{\phi_{\mathrm{i}}+\phi_{\mathrm{tr}}}$ values for different f_{evp} values (ranging from 0.1 to 0.9, with a step of 0.1) are shown in fig. S6. A temperature of 4000 K was assumed for the vapor to calculate the gravitational escape associated with IV (see the following section for different temperatures).

To show the $\frac{\phi_{\mathrm{IS,K}}}{\phi_{\mathrm{IS,K}}+\phi_{\mathrm{IV,K}}}$ and $\frac{\phi_{\mathrm{i}}}{\phi_{\mathrm{i}}+\phi_{\mathrm{tr}}}$ values as a continuous function of f_{evp} varying from 0 to 1, we have constructed a 3D plot (fig. S7) with $\frac{\phi_{\mathrm{IS,K}}}{\phi_{\mathrm{IS,K}}+\phi_{\mathrm{IV,K}}}$ and $\frac{\phi_{\mathrm{i}}}{\phi_{\mathrm{i}}+\phi_{\mathrm{tr}}}$ on the x and y axes and f_{evp} on the z axis. The orange-colored plane in the figure represents the observed K-Rb isotopic slope of 0.172. At 99.99% evaporation, $\frac{\phi_{\mathrm{IS,K}}}{\phi_{\mathrm{IS,K}}+\phi_{\mathrm{IV,K}}}$ falls between 0.25 and 0.29, while $\frac{\phi_{\mathrm{i}}}{\phi_{\mathrm{i}}+\phi_{\mathrm{tr}}}$ can range between 0 and 1.

<u>The effect of gravitational escape temperature on the calculated atmospheric sources and sinks</u>

The above calculations assumed that the gravitational escape associated with IV was thermal escape and happened at a characteristic temperature of 4000 K (*30*, *43*). In reality, the temperature of impacted material could range from ~2500 to 6000 K depending on the impact velocity (*48*, *49*). Here we explore the effect of escape temperature on the calculated relative contributions of the atmospheric sources and sinks.

A higher temperature associated with gravitational escape will lead to more K and Rb loss to space. Consequently, there will be less isotopic difference between the escaped atoms and the remained atoms (fig. S4). Data S1D gives the calculated escaped fractions of K and Rb and the isotopic fractionations between escaped atoms and the bulk vapor released by IV for temperatures between 1000 and 6000 K (Eqs. 1–6), with a step of 500 K. Using those values, we

have calculated the $\frac{\phi_{\mathrm{IS,K}}}{\phi_{\mathrm{IS,K}}+\phi_{\mathrm{IV,K}}}$ and $\frac{\phi_{\mathrm{i}}}{\phi_{\mathrm{i}}+\phi_{\mathrm{tr}}}$ values and the results are shown in fig. S8. The value of $\frac{\phi_{\mathrm{IS,K}}}{\phi_{\mathrm{IS,K}}+\phi_{\mathrm{IV,K}}}$ is always between 0 and 0.35, and the value of $\frac{\phi_{\mathrm{i}}}{\phi_{\mathrm{i}}+\phi_{\mathrm{tr}}}$ is between 0 and 0.53.

The above calculation (fig. S8) used an isotopic fractionation between impact-generated vapor and the bulk lunar soil regolith of −22.25 ‰ for K and −10.18 ‰ for Rb, which are the maximum isotopic fractionation that corresponds to minimal degree of evaporation for K and Rb in the lunar regolith during IV. As discussed above, the isotopic fractionation between vapor and bulk lunar regolith during IV is uncertain and could range from zero to the maximum values depending on the degree of evaporation. Therefore, we have constructed 3D plots to show the effect of degree of evaporation on the calculated $\frac{\phi_{\mathrm{IS,K}}}{\phi_{\mathrm{IS,K}}+\phi_{\mathrm{IV,K}}}$ and $\frac{\phi_{\mathrm{i}}}{\phi_{\mathrm{i}}+\phi_{\mathrm{tr}}}$ values at different temperatures. The result for a temperature of 4000 K is shown in fig. S7. In fig. S9, we show the results for temperatures of 2000, 3000, 5000, and 6000 K. Overall, the value of $\frac{\phi_{\mathrm{IS,K}}}{\phi_{\mathrm{IS,K}}+\phi_{\mathrm{IV,K}}}$ is always between 0 and 0.35. The value of $\frac{\phi_{\mathrm{i}}}{\phi_{\mathrm{i}}+\phi_{\mathrm{tr}}}$ is not highly sensitive to temperature change but depends on the assumed degree of evaporation, and is between 0 and 0.53 for minimal evaporation but can range between 0 and 1 for higher degrees of evaporation (figs. S7–S9).

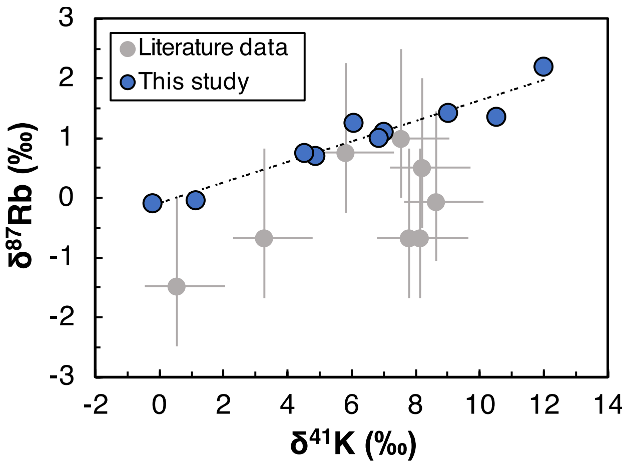

Fig. S1.

Comparison between the Rb and K isotopic compositions of lunar soil samples measured in this study and those reported in literature. The precision of literature data is not sufficient to resolve Rb isotopic variations or the isotopic trend between Rb and K. Blue circles are the measured data from this study while grey circles are literature data (*18–20*). The uncertainties of the lunar soils measured in this study are smaller than the symbols.

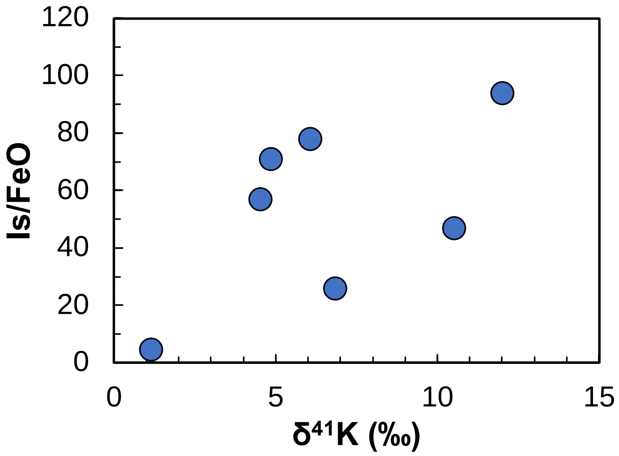

Fig. S2.

Maturity of the lunar soil samples (Is/FeO is used as an indicator, which characterizes the ratio between fine-grained Fe metal and FeO; data from Data S1A) plotted against the measured K isotopic compositions. Soils with heavier K isotopic compositions tend to be more mature. The sizes of the error bars of K isotopic compositions are smaller than the symbols.

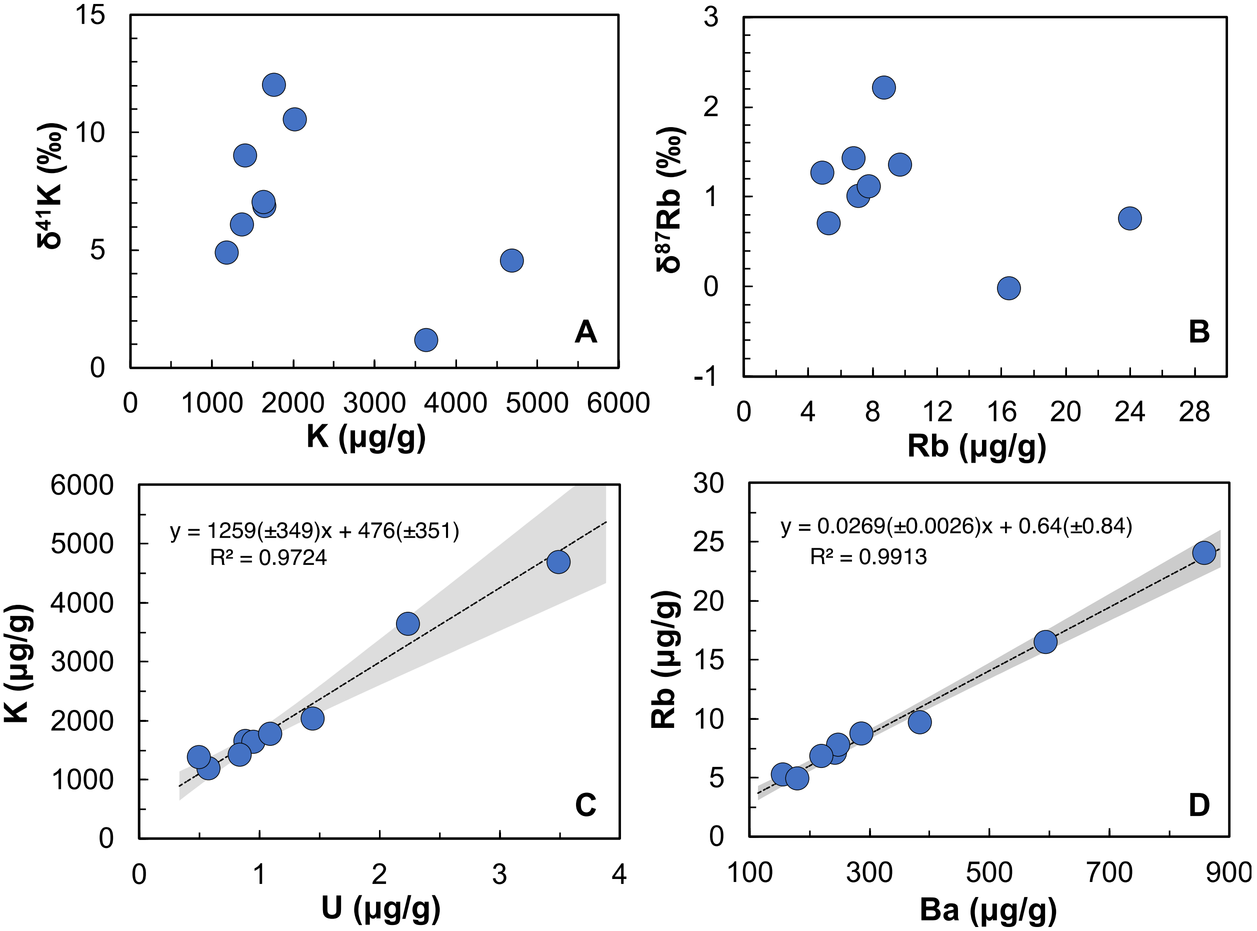

Fig. S3.

Elemental and isotopic compositions of K and Rb in lunar soil samples. (A) The K elemental and isotopic compositions in lunar soils are not correlated. (B) The Rb elemental and isotopic compositions are not correlated. (C) The K and U elemental concentrations show a tight correlation. (D) The Rb and Ba elemental concentrations show a tight correlation. No correlation between elemental and isotopic compositions suggests that they are controlled by different processes. Like K and Rb, elements U and Ba are lithophile and highly incompatible during magmatic differentiation. The elemental correlations between K, Rb, U, and Ba suggest that lunar magmatic differentiation controlled the elemental variations.

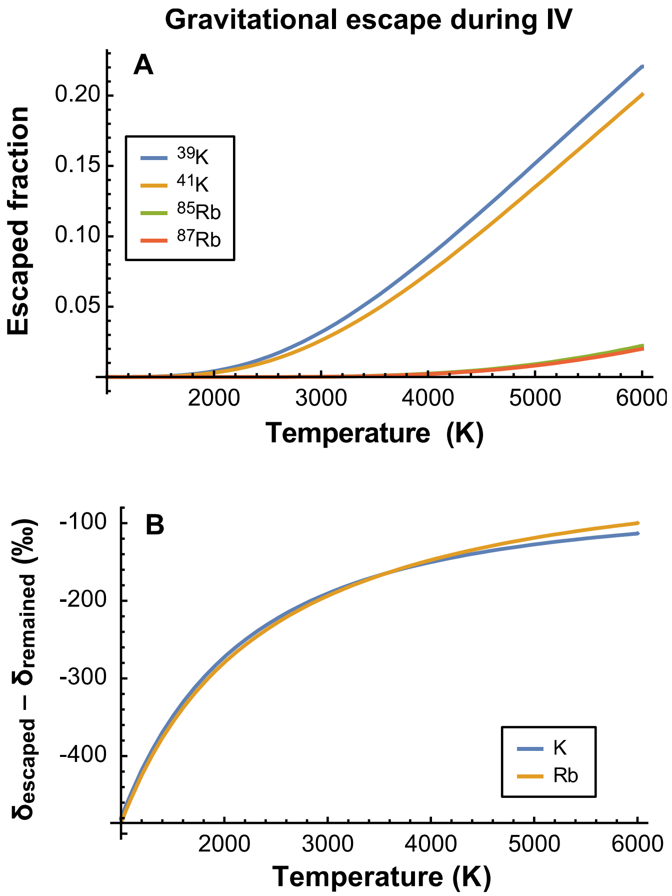

Fig. S4.

Gravitational escape associated with IV. The gravitational escape is considered as thermal escape (Eq. 1) and is a function of temperature. (A) The escaped fractions of K and Rb isotopes among the atoms released by IV as a function of vapor temperature. (B) The isotopic fractionation between the escaped and remained atoms in the vapor as a function of temperature.

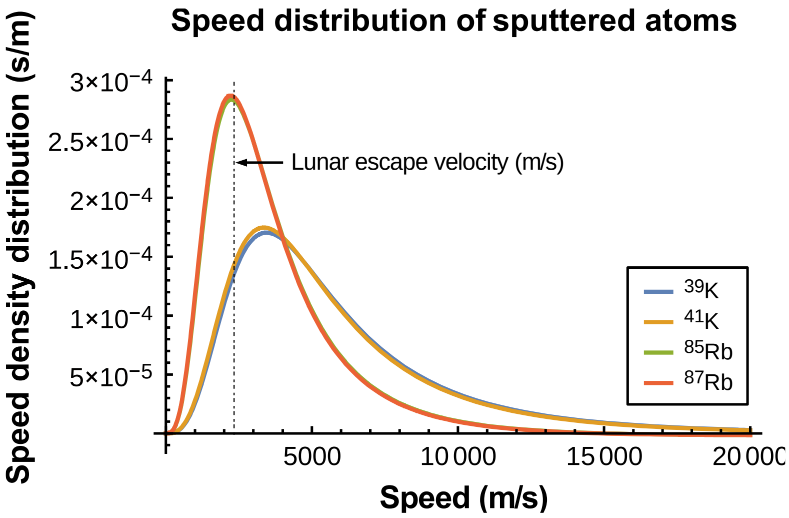

Fig. S5.

The speed distributions of K and Rb isotopes during ion sputtering. Isotopes with speeds higher than the lunar escape velocity (2375 m s^{-1}) are assumed to be lost to space (i.e., gravitational escape).

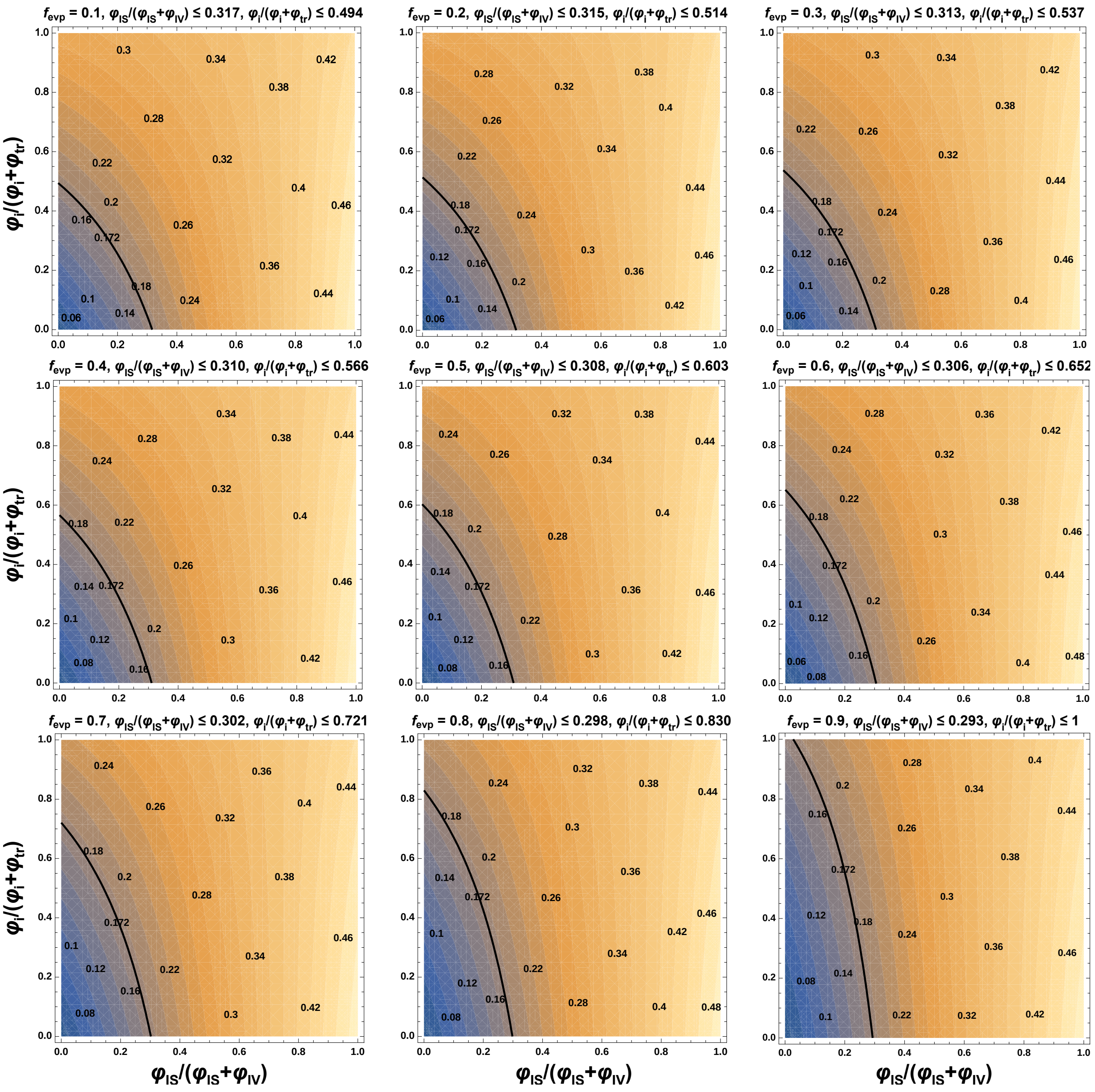

Fig. S6.

The relative contributions of lunar atmospheric sources $\phi_{\mathrm{IS,K}}/(\phi_{\mathrm{IS,K}} + \phi_{\mathrm{IV,K}})$ and sinks $\phi_{\mathrm{i}}/(\phi_{\mathrm{i}} + \phi_{\mathrm{tr}})$ calculated for various degrees of evaporation (f_{evp}; bound between 0 and 1) of lunar soil regolith during IV. Temperature for gravitational escape associated with IV is assumed to be 4000 K. Higher f_{evp} values mean smaller isotopic fractionations between impact vapor and bulk soil regolith. The f_{evp} values used for modeling (0.1–0.9, with a step of 0.1) are shown above each panel, and the calculated values of $\phi_{\mathrm{IS,K}}/(\phi_{\mathrm{IS,K}} + \phi_{\mathrm{IV,K}})$ and $\phi_{\mathrm{i}}/(\phi_{\mathrm{i}} + \phi_{\mathrm{tr}})$ corresponding to the observed K-Rb isotopic slope of 0.172 are also shown above each panel. The thick black lines denote the observed K-Rb isotopic slope of 0.172. The thin lines represent different isotopic slopes, with their respective values labeled on each line.

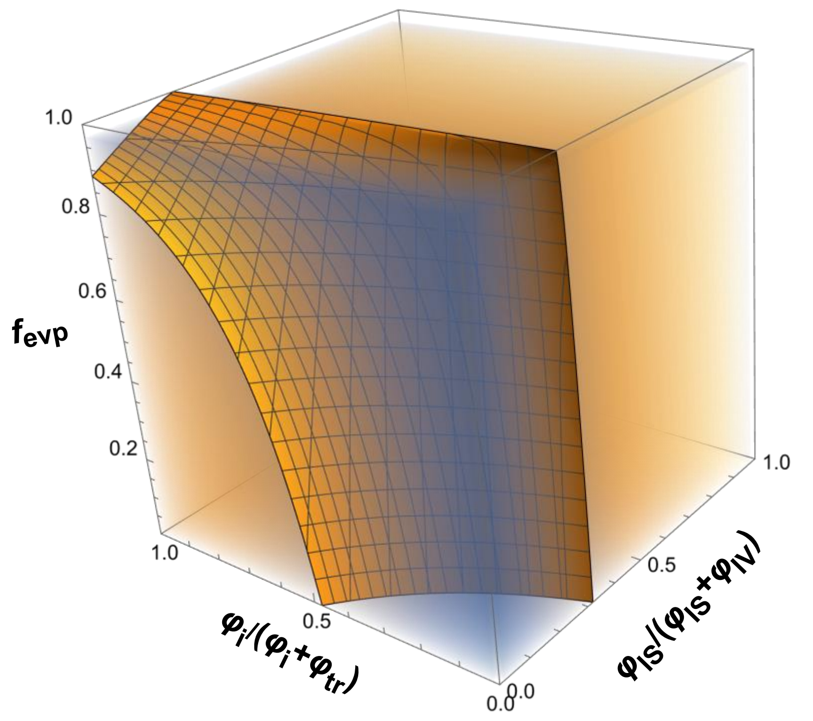

Fig. S7.

A 3D plot of the relative contributions of lunar atmospheric sources $\phi_{\mathrm{IS,K}}/(\phi_{\mathrm{IS,K}} + \phi_{\mathrm{IV,K}})$ and sinks $\phi_{\mathrm{i}}/(\phi_{\mathrm{i}} + \phi_{\mathrm{tr}})$ calculated for different degrees of evaporation (f_{evp}) of elements from the lunar surface during impact vaporization. The degree of evaporation determines the isotopic fractionation between impact vapor and bulk soil regolith, which could range from the maximum to zero with f_{evp} changing from 0 (minimal vaporization) to 1 (quantitative vaporization). The orange-colored surface represents the observed K-Rb isotopic slope in lunar soils of 0.172. Temperature for gravitational escape associated with IV is assumed to be 4000 K.

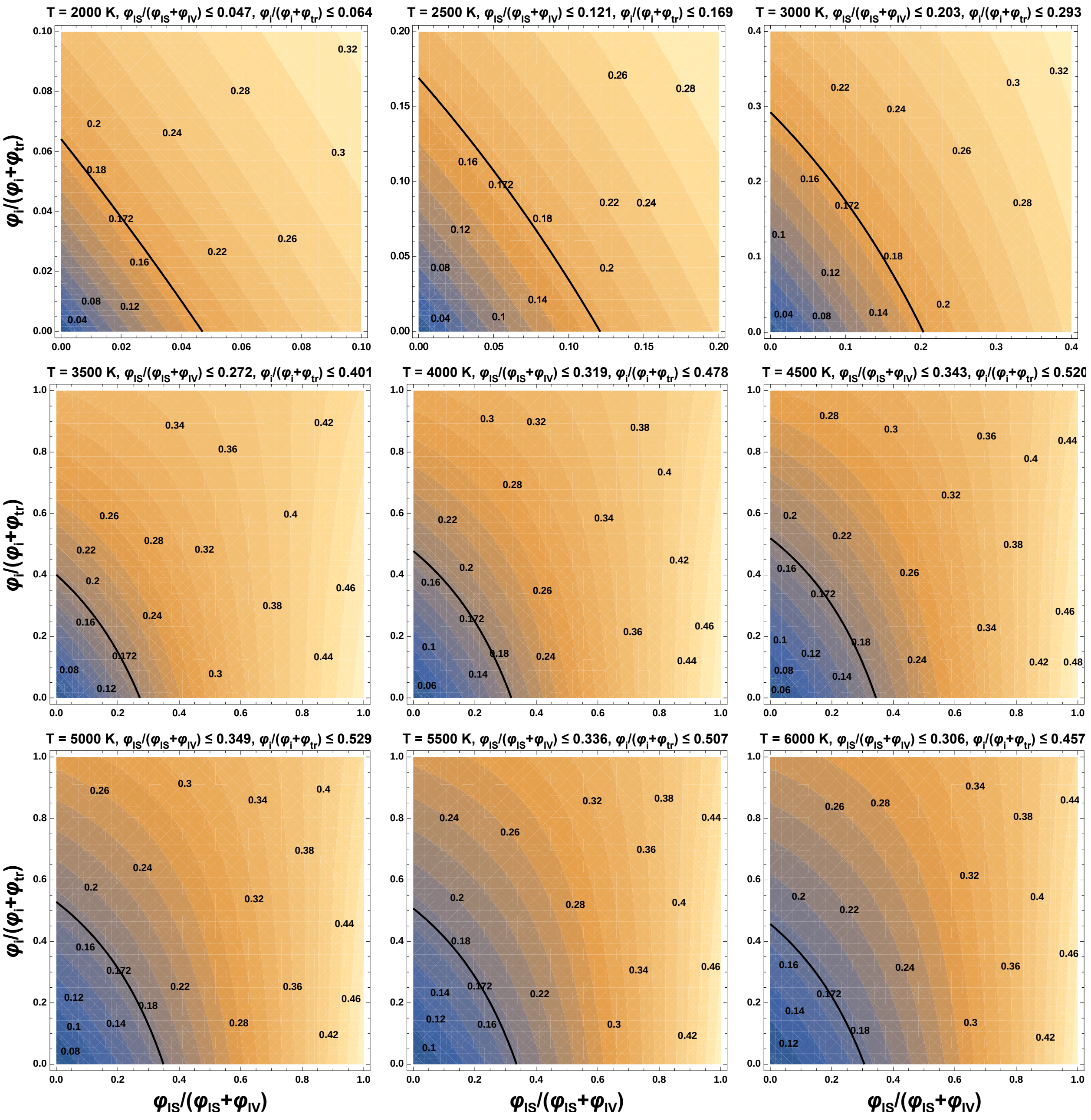

Fig. S8.

The relative contributions of lunar atmospheric sources $\phi_{\mathrm{IS,K}}/(\phi_{\mathrm{IS,K}}+\phi_{\mathrm{IV,K}})$ and sinks $\phi_{\mathrm{i}}/(\phi_{\mathrm{i}}+\phi_{\mathrm{tr}})$ calculated at different temperatures for gravitational escape associated with IV. The vapor-bulk soil isotopic fractionation is assumed to be the maximum, corresponding to minimal degree of evaporation during IV. The temperatures used for modeling and the resulted $\phi_{\mathrm{IS,K}}/(\phi_{\mathrm{IS,K}}+\phi_{\mathrm{IV,K}})$ and sinks $\phi_{\mathrm{i}}/(\phi_{\mathrm{i}}+\phi_{\mathrm{tr}})$ values are all shown above each panel. The thick black lines denote the observed K-Rb isotopic slope of 0.172. The thin lines represent different isotopic slopes, with their respective values labeled on each line.

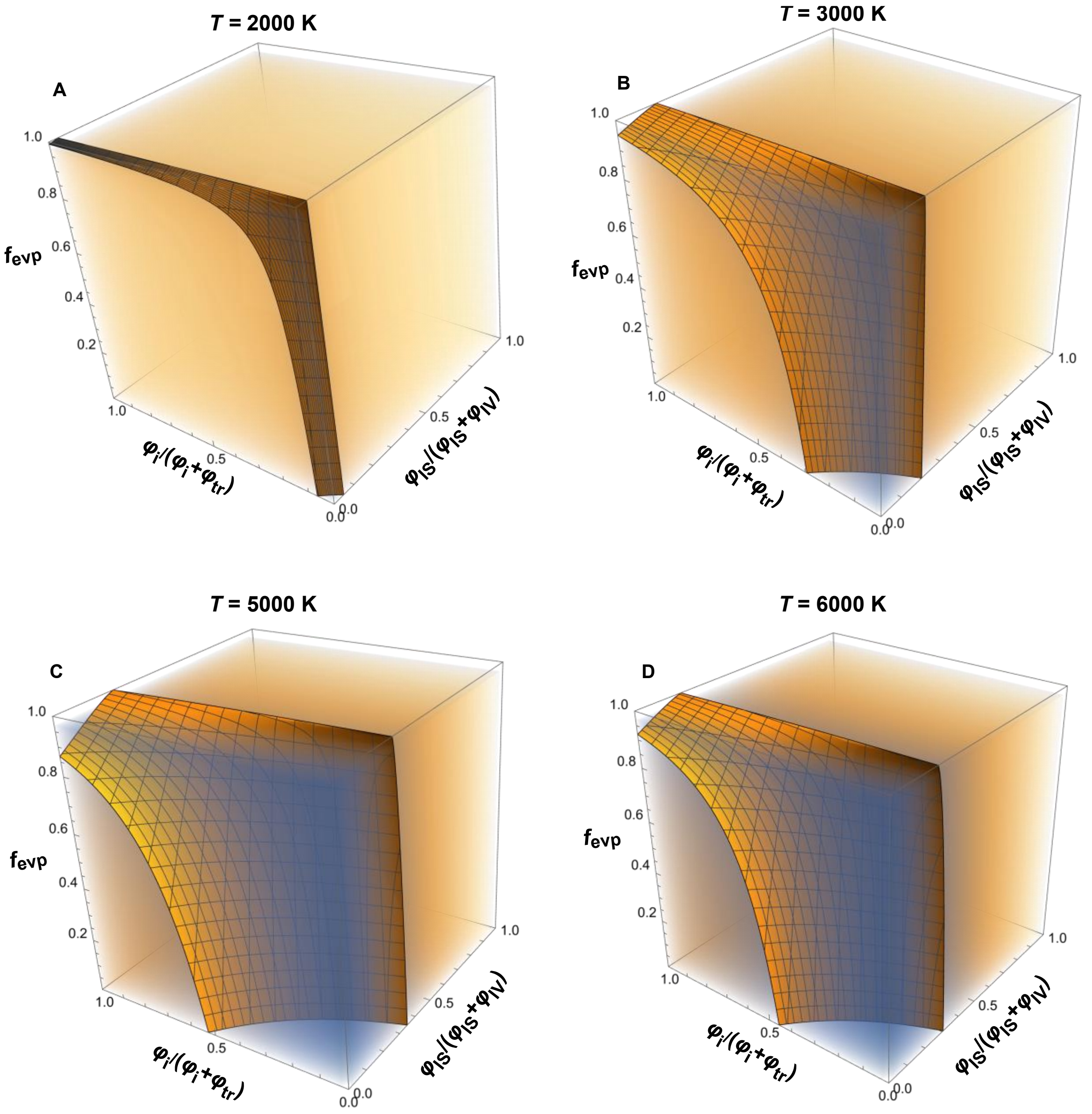

Fig. S9.

3D plots of the relative contributions of lunar atmospheric sources $\phi_{IS,K}/(\phi_{IS,K}+\phi_{IV,K})$ and sinks $\phi_i/(\phi_i+\phi_{tr})$ calculated for different gravitational escape temperatures during IV. For each plot, the assumed temperature is shown above the plot, and the z axis represents the degree of evaporation of elements (f_{evp}) from the lunar surface during IV. The orange-colored surface represents the observed K-Rb isotopic slope of 0.172. (A) At a gravitational escape temperature of 2000 K. (B) At a gravitational escape temperature of 3000 K. (C) At a gravitational escape temperature of 5000 K. (D) At a gravitational escape temperature of 6000 K. See fig. S7 for the result at a gravitational escape temperature of 4000 K.

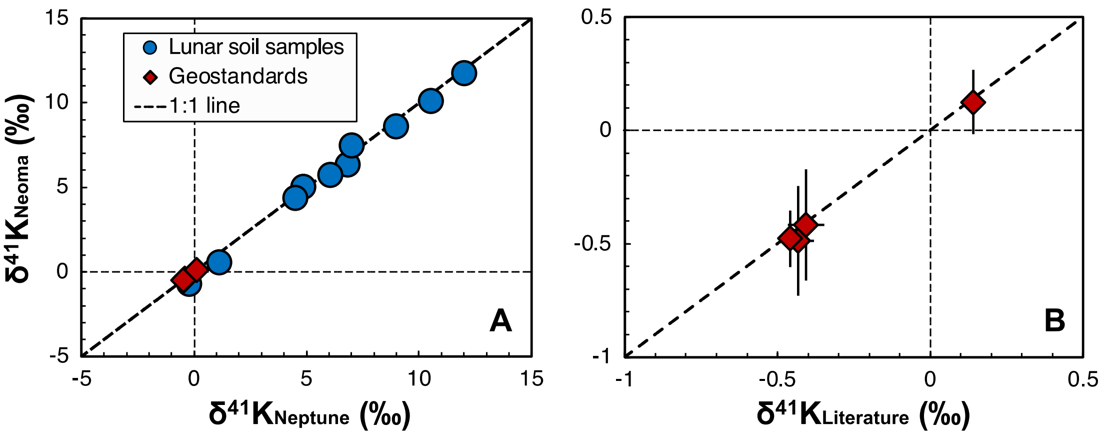

Fig. S10.

Comparison between the K isotopic data obtained with Neoma CC-MC-ICPMS/MS and with Neptune MC-ICPMS and literature. (A) Lunar soil K isotopic compositions obtained with Neoma CC-MC-ICPMS/MS are compared with the data obtained with Neptune (Data S1F and G). All lunar soil data are from this study. Geostandands were measured with Neoma MS/MS (Data S1G), and are plotted against literature data. The data follow the 1:1 line, suggesting agreement between the two methods. Uncertainties are smaller than the symbols. (B) A close-up view of the geostandards in (A).

Data S1. (separate file)

Data S1A. Isotopic compositions of K and Rb and concentrations of selected elements in lunar soils and reference samples (*82*).
Data S1B. The K, Rb, and Li isotopic compositions of lunar soil samples from literature (*18–20*, *83*, *84*).
Data S1C. Parameters and values used in the modeling of lunar soil K and Rb isotopic evolution (*30*, *32*, *39–44*, *48*, *49*, *79*, *80*).
Data S1D. The K and Rb escaped fractions and isotope fractionations for gravitational escape at various temperatures during impact vaporization.
Data S1E. Operating parameters of the Neoma MS/MS and the Apex Omega introduction system.
Data S1F. Isotopic compositions of lunar soil samples measured with Neoma CC-MC-ICP-MS/MS.
Data S1G. Isotopic compositions of geostandards measured with Neoma CC-MC-ICP-MS/MS (*59*, *60*, *66*, *71*).

REFERENCES AND NOTES

1. D. M. Hunten, A. L. Sprague, Origin and character of the lunar and mercurian atmospheres. *Adv. Space Res.* **19**, 1551–1560 (1997).

2. R. M. Killen, W.-H. Ip, The surface-bounded atmospheres of Mercury and the Moon. *Rev. Geophys.* **37**, 361–406 (1999).

3. A. Potter, T. Morgan, Discovery of sodium and potassium vapor in the atmosphere of the Moon. *Science* **241**, 675–680 (1988).

4. A. L. Tyler, R. W. Kozlowski, D. M. Hunten, Observations of sodium in the tenuous lunar atmosphere. *Geophys. Res. Lett.* **15**, 1141–1144 (1988).

5. S. M. Smith, J. K. Wilson, J. Baumgardner, M. Mendillo Discovery of the distant lunar sodium tail and its enhancement following the Leonid Meteor Shower of 1998. *Geophys. Res. Lett.* **26**, 1649–1652 (1999).

6. S. A. Stern, The lunar atmosphere: History, status, current problems, and context. *Rev. Geophys.* **37**, 453–491 (1999).

7. W. H. Smyth, M. L. Marconi, Theoretical overview and modeling of the sodium and potassium atmospheres of the Moon. *Astrophys. J.* **443**, 371–392 (1995).

8. J. R. Szalay, M. Horányi, A. Colaprete, M. Sarantos Meteoritic influence on sodium and potassium abundance in the lunar exosphere measured by LADEE. *Geophys. Res. Lett.* **43**, 6096–6102 (2016).

9. A. Colaprete, M. Sarantos, D. H. Wooden, T. J. Stubbs, A. M. Cook, M. Shirley How surface composition and meteoroid impacts mediate sodium and potassium in the lunar exosphere. *Science* **351**, 249–252 (2016).

10. A. L. Sprague, R. W. H. Kozlowski, D. M. Hunten, W.K. Wells, F. A. Grosse The sodium and potassium atmosphere of the moon and its interaction with the surface. *Icarus* **96**, 27–42 (1992).

11. R. M. Killen, T.H. Morgan, A.E. Potter, G. Bacon, I. Ajang, A. R. Poppe Coronagraphic observations of the lunar sodium exosphere 2018–2019. *Icarus* **355**, 114155 (2021).

12. B. Fegley Jr, K. Lodders, N. S. Jacobson, Chemical equilibrium calculations for bulk silicate earth material at high temperatures. *Geochemistry* **83**, 125961 (2023).

13. K. Wang, S. B. Jacobsen, Potassium isotopic evidence for a high-energy giant impact origin of the Moon. *Nature* **538**, 487–490 (2016).

14. N. X. Nie, N. Dauphas, Vapor drainage in the protolunar disk as the cause for the depletion in volatile elements of the Moon. *Astrophys. J.* **884**, L48 (2019).

15. E. A. Pringle, F. Moynier, Rubidium isotopic composition of the Earth, meteorites, and the Moon: Evidence for the origin of volatile loss during planetary accretion. *Earth Planet. Sci. Lett.* **473**, 62–70 (2017).

16. N. X. Nie, X. Y. Chen, Z. J. Zhang, J. Y. Hu, W. Liu, F. L. H. Tissot, F. Z. Teng, A. Shahar, N. Dauphas Rubidium and potassium isotopic variations in chondrites and Mars: Accretion signatures and planetary overprints. *Geochim. Cosmochim. Acta* **344**, 207–229 (2023).

17. Z. Tian, B. L. Jolliff, R. L. Korotev, B. Fegley Jr, K. Lodders, J. M. D. Day, H. Chen, K. Wang Potassium isotopic composition of the Moon. *Geochim. Cosmochim. Acta* **280**, 263–280 (2020).

18. E. L. Garner, L. A. Machlan, I. L. Barnes, The isotopic composition of lithium, potassium, and rubidium in some Apollo 11, 12, 14, 15, and 16 samples, in *Proceedings of the Sixth Lunar Science Conference* (Houston, Texas, 17 to 21 March 1975), pp. 1845–1855.

19. S. E. Church, G. R. Tilton, J. E. Wright, C.-N. Lee-Hu, Volatile element depletion and K-39/K-41 fractionation in lunar soils, in *Proceeding of the Seventh Lunar Science Conference* (Houston, Texas, 15 to 19 March 1976), pp. 423–439.

20. M. Humayun, R. N. Clayton, Precise determination of the isotopic composition of potassium: Application to terrestrial rocks and lunar soils. *Geochim. Cosmochim. Acta* **59**, 2115–2130 (1995).

21. N. X. Nie, N. Dauphas, T. Hopp, J. Y. Hu, Z. J. Zhang, R. Yokochi, T. J. Ireland, F. L. H. Tissot, Chromatography purification of Rb for accurate isotopic analysis by MC-ICPMS: A comparison between AMP-PAN, cation-exchange, and Sr resins. *J. Anal. At. Spectrom.* **36**, 2588–2602 (2021).

22. R. V. Morris, Surface exposure indices of lunar soils-A comparative FMR study, in *Proceedings of the Seventh Lunar Science Conference, Houston, Texas, 15 to 19 March 1976* (Pergamon Press Inc., New York, 1976), vol. 1, pp. 315–335.

23. N. Dauphas, N. X. Nie, M. Blanchard, Z. J. Zhang, H. Zeng, J. Y. Hu, M. Meheut, C. Visscher, R. Canup, T. Hopp, The extent, nature, and origin of K and Rb depletions and isotopic fractionations in Earth, the Moon, and other planetary bodies. *Planet. Sci. J.* **3**, 29 (2022).

24. B. V. Yakshinskiy, T. E. Madey, Photon-stimulated desorption as a substantial source of sodium in the lunar atmosphere. *Nature* **400**, 642–644 (1999).

25. B. V. Yakshinskiy, T. E. Madey, Desorption induced by electronic transitions of Na from SiO2: Relevance to tenuous planetary atmospheres. *Surf. Sci.* **451**, 160–165 (2000).

26. B. V. Yakshinskiy, T. E. Madey, Electron- and photon-stimulated desorption of K from ice surfaces. *J. Geophys. Res. Planets* **106**, 33303–33307 (2001).

27. B. V. Yakshinskiy, T. E. Madey, Photon-stimulated desorption of Na from a lunar sample: Temperature-dependent effects. *Icarus* **168**, 53–59 (2004).

28. M. Sarantos, R. M. Killen, A. Surjalal Sharma, J. A. Slavin, Sources of sodium in the lunar exosphere: Modeling using ground-based observations of sodium emission and spacecraft data of the plasma. *Icarus* **205**, 364–374 (2010).

29. M. Kagitani, M. Taguchi, A. Yamazaki, I. Yoshikawa, G. Murakami, K. Yoshioka, S. Kameda, S. Okano, Variation in lunar sodium exosphere measured from lunar orbiter SELENE (Kaguya). *Planet. Space Sci.* **58**, 1660–1664 (2010).

30. P. Wurz, S. Fatemi, A. Galli, J. Halekas, Y. Harada, N. Jäggi, J. Jasinski, H. Lammer, S. Lindsay, M. N. Nishino, T. M. Orlando, J. M. Raines, M. Scherf, J. Slavin, A. Vorburger, R. Winslow, Particles and photons as drivers for particle release from the surfaces of the Moon and Mercury. *Space Sci. Rev.* **218**, 10 (2022).

31. P. K. Haff, Z. E. Switkowski, D. S. Burnett, T. A. Tombrello, Gravitational and recoil contributions to surface mass fractionation by solar-wind sputtering, in *Proceeding of the 8th Lsunar Science Conference, Houston, Texas, 14 to 18March 1977* (Pergamon Press Inc., New York, 1977), pp. 3807–3815.

32. R. M. Housley, A model for chemical and isotopic fractionation in the lunar regolith by impact vaporization, in *Proceedings of the 10th Lunar and Planetary Science Conference, Houston, Texas, 19 to 23 March 1979* (Pergamon, Press Inc., 1979), vol. 2, pp. 1673–1683.

33. T. H. Morgan, H. A. Zook, A. E. Potter, Production of sodium vapor from exposed regolith in the inner solar system, in *Proceedingas of the 19th Lunar and Planetary Science Conference, Houston, Texas, 14 to 18 March 1988,* (Cambridge/Houston, Texas, Cambridge Univ. Press/Lunar and Planetary Institute, 1989), pp. 297–304.

34. R. Clayton, T. Mayeda, J. Hurd, Loss of oxygen, silicon, sulfur, and potassium from the lunar regolith, in *Proceedings of the 5th Lunar and Planetary Science Conference Houston, Texas, 18 to 22 March 1974* (Pergamon Press Inc., New York, 1974), vol. 2, pp. 1801–1809.

35. Z. L. Liau, W. L. Brown, R. Homer, J. M. Poate, Surface-layer composition changes in sputtered alloys and compounds. *Appl. Phys. Lett.* **30**, 626–628 (1977).

36. W. A. Russell, D. A. Papanastassiou, T. A. Tombrello, The fractionation of Ca isotopes by sputtering. *Radiat. Eff.* **52**, 41–52 (1980).

37. Y. Yu, R. H. Hewins, C. M. O. ’D. Alexander, J. Wang, Experimental study of evaporation and isotopic mass fractionation of potassium in silicate melts. *Geochim. Cosmochim. Acta* **67**, 773–786 (2003).

38. F. M. Richter, R. A. Mendybaev, J. N. Christensen, D. Ebel, A. Gaffney, Laboratory experiments bearing on the origin and evolution of olivine-rich chondrules. *Meteorit. Planet. Sci.* **46**, 1152–1178 (2011).

39. Z. J. Zhang, N. X. Nie, R. A. Mendybaev, M.-C. Liu, J. J. Hu, T. Hopp, E. E. Alp, B. Lavina, E. S. Bullock, K. D. McKeegan, Loss and isotopic fractionation of alkali elements during diffusion-limited evaporation from molten silicate: Theory and experiments. *ACS Earth Space Chem.* **5**, 755–784 (2021).

40. R. L. Hervig, F. K. Mazdab, P. Williams, Y. Guan, G. R. Huss, L. A. Leshin, Useful ion yields for Cameca IMS 3f and 6f SIMS: Limits on quantitative analysis. *Chem. Geol.* **227**, 83–99 (2006).

41. P. Sigmund, Theory of sputtering. I. Sputtering yield of amorphous and polycrystalline targets. *Phys. Rev.* **184**, 383–416 (1969).

42. P. Sigmund, Mechanisms and theory of physical sputtering by particle impact. *Nucl. Instrum. Methods Phys. Res. B* **27**, 1–20 (1987).

43. P. Wurz, J. A. Whitby, U. Rohner, J. A. Martín-Fernández, H. Lammer, C. Kolb, Self-consistent modelling of Mercury’s exosphere by sputtering, micro-meteorite impact and photon-stimulated desorption. *Planet. Space Sci.* **58**, 1599–1616 (2010).

44. D. Gamborino, P. Wurz, Velocity distribution function of Na released by photons from planetary surfaces. *Planet. Space Sci.* **159**, 97–104 (2018).

45. R. Manka, F. Michel, Lunar atmosphere as a source of argon-40 and other lunar surface elements. *Science* **169**, 278–280 (1970).

46. A. E. Potter, T. H. Morgan, Variation of lunar sodium emission intensity with phase angle. *Geophys. Res. Lett.* **21**, 2263–2266 (1994).

47. G. Herzog, F. Moynier, F. Albarède, A. Berezhnoy, Isotopic and elemental abundances of copper and zinc in lunar samples, Zagami, Pele's hairs, and a terrestrial basalt. *Geochim. Cosmochim. Acta* **73**, 5884–5904 (2009).

48. G. Eichhorn, Impact light flash studies: Temperature, ejecta, vaporization, in *Interplanetary Dust and Zodiacal Light*. *Lecture Notes in Physics,* H. Elsässer, H. Fechting, Eds. (Springer, Berlin, Heidelberg, 1976), pp. 243–247.

49. G. Eichhorn, Heating and vaporization during hypervelocity particle impact. *Planet. Space Sci.* **26**, 463–467 (1978).

50. S. Epstein, H. P. Taylor Jr, Investigation of the carbon, hydrogen, oxygen, and silicon isotope and concentration relationships on the grain surfaces of a variety of lunar soils and in some Apollo 15 and 16 core samples, in *Proceedings of the 6th Lunar Science Conference, Houston, Texas, 17 to 21 March 1975,* (Pergamon Press Inc., New York, 1975), vol. 2, pp. 1771–1798.

51. D. G. Sands, K. J. R. Rosman, J. R. de Laeter, A preliminary study of cadmium mass fractionation in lunar soils. *Earth Planet. Sci. Lett.* **186**, 103–111 (2001).

52. R. A. Wiesli, B. L. Beard, L. A. Taylor, C. M. Johnson, Space weathering processes on airless bodies: Fe isotope fractionation in the lunar regolith. *Earth Planet. Sci. Lett.* **216**, 457–465 (2003).

53. F. Moynier, F. Albarède, G. F. Herzog, Isotopic composition of zinc, copper, and iron in lunar samples. *Geochim. Cosmochim. Acta* **70**, 6103–6117 (2006).

54. K. Wang, F. Moynier, F. A. Podosek, J. Foriel, An iron isotope perspective on the origin of the nanophase metallic iron in lunar regolith. *Earth Planet. Sci. Lett.* **337-338**, 17–24 (2012).

55. V. Tenishev, M. Rubin, O. J. Tucker, M. R. Combi, M. Sarantos, Kinetic modeling of sodium in the lunar exosphere. *Icarus* **226**, 1538–1549 (2013).

56. M. Sarantos, S. Tsavachidis, The boundary of alkali surface boundary exospheres of Mercury and the Moon. *Geophys. Res. Lett.* **47**, e2020GL088930 (2020).

57. N. X. Nie, X.-Y. Chen, T. Hopp, J. Y. Hu, Z. J. Zhang, F.-Z. Teng, A. Shahar, N. Dauphas, Imprint of chondrule formation on the K and Rb isotopic compositions of carbonaceous meteorites. *Sci. Adv.* **7**, eabl3929 (2021).

58. N. X. Nie, D. Wang, Z. A. Torrano, R. W. Carlson, C. M. O'D. Alexander, A. Shahar, Meteorites have inherited nucleosynthetic anomalies of potassium-40 produced in supernovae. *Science* **379**, 372–376 (2023).

59. H. Chen, Z. Tian, B. Tuller-Ross, R. L. Korotev, K. Wang, High-precision potassium isotopic analysis by MC-ICP-MS: An inter-laboratory comparison and refined K atomic weight. *J. Anal. At. Spectrom* **34**, 160–171 (2019).

60. Y. Hu, X.-Y. Chen, Y.-K. Xu, F.-Z. Teng, High-precision analysis of potassium isotopes by HR-MC-ICPMS. *Chem. Geol.* **493**, 100–108 (2018).

61. W. Li, B. L. Beard, S. Li, Precise measurement of stable potassium isotope ratios using a single focusing collision cell multi-collector ICP-MS. *J. Anal. At. Spectrom* **31**, 1023–1029 (2016).

62. W. Li, M. Cui, Q. Pan, J. Wang, B. Gao, S. Liu, M. Yuan, B. Su, Y. Zhao, F.-Z. Teng, High-precision potassium isotope analysis using the Nu Sapphire collision cell (CC)-MC-ICP-MS. *Sci. China Earth Sci.*, **65**, 1510–1521 (2022).

63. K. Wang (王昆), S. B. Jacobsen, An estimate of the Bulk Silicate Earth potassium isotopic composition based on MC-ICPMS measurements of basalts. *Geochim. Cosmochim. Acta* **178**, 223–232 (2016).

64. Y. Ku, S. B. Jacobsen, Potassium isotope anomalies in meteorites inherited from the protosolar molecular cloud. *Sci. Adv.* **6**, eabd0511 (2020).

65. H. Chen, N. J. Saunders, M. Jerram, A. N. Halliday, High-precision potassium isotopic measurements by collision cell equipped MC-ICPMS. *Chem. Geol.*, **578**, 120281 (2021).

66. F. Moynier, Y. Hu, K. Wang, Y. Zhao, Y. Gérard, Z. Deng, J. Moureau, W. Li, J. I. Simon, F.-Z. Teng, Potassium isotopic composition of various samples using a dual-path collision cell-capable multiple-collector inductively coupled plasma mass spectrometer Nu instruments Sapphire. *Chem. Geol.* **571**, 120144 (2021).

67. X.-Y. Zheng, X.-Y. Chen, W. Ding, Y. Zhang, S. Charin, Y. Gérard, High precision analysis of stable potassium (K) isotopes by the collision cell MC-ICP-MS "Sapphire" and a correction method for concentration mismatch. *J. Anal. At. Spectrom* **37**, 1273–1287 (2022).

68. P. Télouk, E. Albalat, T. Tacail, F. Arnaud-Godet, V. Balter, Steady analyses of potassium stable isotopes using a Thermo Scientific Neoma MC-ICP-MS. *J. Anal. At. Spectrom* **37**, 1259–1264 (2022).

69. G. Craig, H. Wehrs, D. G. Bevan, M. Pfeifer, J. Lewis, C. D. Coath, T. Elliott, C. Huang, N. S. Lloyd, J. B. Schwieters, Project vienna: A novel precell mass filter for collision/reaction cell MC-ICPMS/MS. *Anal. Chem.* **93**, 10519–10527 (2021).

70. N. Dauphas, T. Hopp, G. Craig, Z. J. Zhang, M. C. Valdes, P. R. Heck, B. L. A. Charlier, E. A. Bell, T. Mark Harrison, A. M. Davis, L. Dussubieux, P. R. Williams, M. J. Krawczynski, C. Bouman, N. S. Lloyd, D. Tollstrup, J. B. Schwieters, In situ ^{87}Rb–^{87}Sr analyses of terrestrial and extraterrestrial samples by LA-MC-ICP-MS/MS with double Wien filter and collision cell technologies. *J. Anal. At. Spectrom* **37**, 2420–2441 (2022).

71. Y.-K. Xu, Y. Hu, X.-Y. Chen, T.-Y. Huang, R. S. Sletten, D. Zhu, F.-Z. Teng, Potassium isotopic compositions of international geological reference materials. *Chem. Geol.* **513**, 101–107 (2019).

72. F. M. Richter, Timescales determining the degree of kinetic isotope fractionation by evaporation and condensation. *Geochim. Cosmochim. Acta* **68**, 4971–4992 (2004).

73. N. Dauphas, F. Poitrasson, C. Burkhardt, H. Kobayashi, K. Kurosawa, Planetary and meteoritic Mg/Si and δ30Si variations inherited from solar nebula chemistry. *Earth Planet. Sci. Lett.* **427**, 236–248 (2015).

74. F. M. Richter, P. E. Janney, R. A. Mendybaev, A. M. Davis, M. Wadhwa, Elemental and isotopic fractionation of type B CAI-like liquids by evaporation. *Geochim. Cosmochim. Acta* **71**, 5544–5564 (2007).

75. F. M. Richter, A. M. Davis, D. S. Ebel, A. Hashimoto, Elemental and isotopic fractionation of type B calcium-, aluminum-rich inclusions: Experiments, theoretical considerations, and constraints on their thermal evolution. *Geochim. Cosmochim. Acta* **66**, 521–540 (2002).

76. G. De Maria, G. Balducci, M. Guido, V. Piacente, Mass spectrometric investigation of the vaporization process of Apollo 12 lunar samples. *Proc. Lunar Sci. Conf.* **2**, 1367–1380 (1971).

77. J. J. Naughton, J. V. Derby, V. A. Lewis, Vaporization from heated lunar samples and the investigation of lunar erosion by volatilized alkalis. *Proc. Lunar Sci. Conf.* **2**, 449–457 (1971).

78. E. K. Gibson Jr, N. J. Hubbard, Thermal volatilization studies on lunar samples. *Proc. Lunar Sci. Conf.* **3**, 2003–2014 (1972).

79. Y. Kudriavtsev, A. Villegas, A. Godines, R. Asomoza, Calculation of the surface binding energy for ion sputtered particles. *Appl. Surf. Sci.* **239**, 273–278 (2005).

80. P. Wurz, U. Rohner, J. A. Whitby, C. Kolb, H. Lammer, P. Dobnikar, J. A. Martín-Fernández, The lunar exosphere: The sputtering contribution. *Icarus* **191**, 486–496 (2007).

81. A. M. Davis, Volatile evolution and loss, in *Meteorites and the early solar system II* (University of Arizona Press, 2006), p. 295–307.

82. R. V. Morris, The surface exposure/maturity/of lunar soils—Some concepts and $I_{s/FeO}$ compilation, in *Proceedings of the 9th Lunas and Planetary Science Conference, Houston, Texas, 13 to 17 March 1978* (Pergamon Press Inc., 1978) vol. 2, pp. 2287–2297.

83. T. Magna, U. Wiechert, A. N. Halliday, New constraints on the lithium isotope compositions of the Moon and terrestrial planets. *Earth Planet. Sci. Lett.* **243**, 336–353 (2006).

84. I. Barnes, E. Garner, J. Gramlich, L. Machlan, J. Moody, L. Moore, T. Murphy, W. Shields, Isotopic abundance ratios and concentrations of selected elements in some Apollo 15 and Apollo 16 samples. *Proc. Lunar Sci. Conf.*, **4**, 1197–1207 (1973).
